# Glycoengineered Monoclonal Antibodies with Homogeneous Glycan (M3, G0, G2, and A2) Using a Chemoenzymatic Approach Have Different Affinities for FcγRIIIa and Variable Antibody-Dependent Cellular Cytotoxicity Activities

**DOI:** 10.1371/journal.pone.0132848

**Published:** 2015-07-22

**Authors:** Masaki Kurogochi, Masako Mori, Kenji Osumi, Mami Tojino, Shu-ichi Sugawara, Shou Takashima, Yuriko Hirose, Wataru Tsukimura, Mamoru Mizuno, Junko Amano, Akio Matsuda, Masahiro Tomita, Atsushi Takayanagi, Shin-Ichiro Shoda, Takashi Shirai

**Affiliations:** 1 Laboratory of Glycobiology, The Noguchi Institute, 1-8-1 Kaga, Itabashi-ku, Tokyo, Japan; 2 Laboratory of Glyco-Bioengineering, The Noguchi Institute, 1-8-1 Kaga, Itabashi-ku, Tokyo, Japan; 3 Laboratory of Glyco-organic Chemistry, The Noguchi Institute, 1-8-1 Kaga, Itabashi-ku, Tokyo, Japan; 4 Immuno-Biological Laboratories Co., Ltd., 1091-1 Naka, Fujioka-shi, Gunma, Japan; 5 Department of Molecular Biology, Keio University School of Medicine, 35 Shinanomachi, Shinjuku-ku, Tokyo, Japan; 6 Graduate School of Engineering, Tohoku University, Aoba-ku, Sendai, Japan; National Cancer Institute, NIH, UNITED STATES

## Abstract

Many therapeutic antibodies have been developed, and IgG antibodies have been extensively generated in various cell expression systems. IgG antibodies contain *N*-glycans at the constant region of the heavy chain (Fc domain), and their *N*-glycosylation patterns differ during various processes or among cell expression systems. The Fc *N*-glycan can modulate the effector functions of IgG antibodies, such as antibody-dependent cellular cytotoxicity (ADCC) and complement dependent cytotoxicity (CDC). To control Fc *N*-glycans, we performed a rearrangement of Fc *N*-glycans from a heterogeneous *N*-glycosylation pattern to homogeneous *N*-glycans using chemoenzymatic approaches with two types of endo-β-*N*-acetyl glucosaminidases (ENG’ases), one that works as a hydrolase to cleave all heterogeneous *N*-glycans, another that is used as a glycosynthase to generate homogeneous *N*-glycans. As starting materials, we used an anti-Her2 antibody produced in transgenic silkworm cocoon, which consists of non-fucosylated pauci-mannose type (Man_2-3_GlcNAc_2_), high-mannose type (Man_4-9_GlcNAc_2_), and complex type (Man_3_GlcNAc_3-4_) *N*-glycans. As a result of the cleavage of several ENG’ases (endoS, endoM, endoD, endoH, and endoLL), the heterogeneous glycans on antibodies were fully transformed into homogeneous-GlcNAc by a combination of endoS, endoD, and endoLL. Next, the desired *N*-glycans (M3; Man_3_GlcNAc_1_, G0; GlcNAc_2_Man_3_GlcNAc_1_, G2; Gal_2_GlcNAc_2_Man_3_GlcNAc_1_, A2; NeuAc_2_Gal_2_GlcNAc_2_Man_3_GlcNAc_1_) were transferred from the corresponding oxazolines to the GlcNAc residue on the intact anti-Her2 antibody with an ENG’ase mutant (endoS-D233Q), and the glycoengineered anti-Her2 antibody was obtained. The binding assay of anti-Her2 antibody with homogenous *N*-glycans with FcγRIIIa-V158 showed that the glycoform influenced the affinity for FcγRIIIa-V158. In addition, the ADCC assay for the glycoengineered anti-Her2 antibody (mAb-M3, mAb-G0, mAb-G2, and mAb-A2) was performed using SKBR-3 and BT-474 as target cells, and revealed that the glycoform influenced ADCC activity.

## Introduction

**Fig 1:**
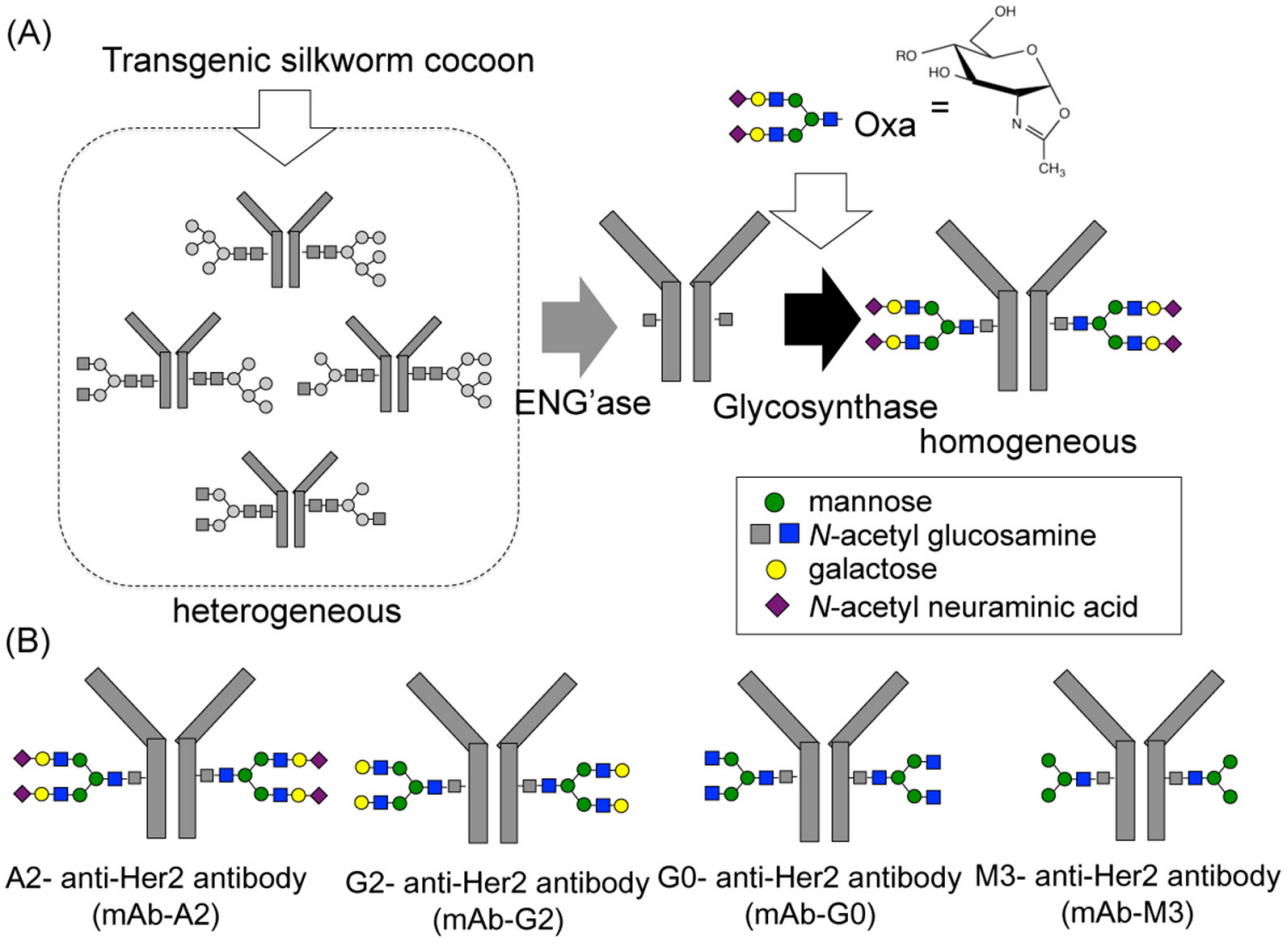
(A) Protocol of chemoenzymatic synthesis using ENG’ase and glycosynthase. (B) Diagram of the homogeneous glycosylated anti-Her2 mAb with M3 (mAb-M3), G0 (mAb-G0), G2 (mAb-G2), and A2 (mAb-A2).

Monoclonal antibodies (mAbs) of IgG have become important therapeutic agents for numerous diseases such as cancer, autoimmune, and infectious diseases [1, 2, 3]. IgG antibodies are composed of two heavy chains and two light chains, which can be divided into two regions based on amino acid sequence variability: the fragment antigen binding (Fab) region can recognize specific antigens, while the fragment crystallizable (Fc) region plays a role in modulating immune cell activity, such as antibody-dependent cellular cytotoxicity (ADCC) and complement-dependent cytotoxicity (CDC) [4, 5]. *N*-glycans attached at a single conserved site within the Fc region are critical for the antibody’s effector functions. In addition, X-ray crystallographic [6, 7] and NMR structural studies [8, 9, 10] have shown that the Fc *N*-glycans are located within the CH2 domain of each heavy chain and have multiple noncovalent interactions with the Fc domain to retain its conformational flexibility [11, 12, 13]. Thus, glycosylation is a primary concern in the biopharmaceutical industry, and transgenic cell lines (mammalian [14, 15, 16], insect [17], yeast [18], plant cells [19], etc.) [20] have been engineered to generate antibody products with depleted core fucose and lacking galactosylated and sialylated extensions, because these cell lines generally produce glycoproteins with non-human glycoforms (terminal Galα1-3Gal, NeuGc epitope, etc) affecting the immunogenic response. However, due to the action of many endogenous glyco-related factors (glycosidase, glycosyltransferase, nucleotide sugar, protein trafficking, etc), it is difficult to generate mAbs with homogeneous glycans using a cell expression system. Recently, therapeutic mAbs with the ability to express defined Fc *N*-glycans have been developed using several targeted gene knockdown or knockout approaches in different cell lines [14, 18, 21]. An alternative approach to modulate the heterogeneity of glycosylation in glycoproteins is to perform glycosylation remodeling by trimming the heterogeneous *N*-glycans and extending the oligosaccharide moiety through enzymatic glycosylation, as shown in Fig 1A. Wang *et al*. reported a chemoenzymatic approach for glycosylation remodeling of intact full-length mAbs (rituximab produced by CHO cells) that takes advantage of the transglycosylation activity of endoS and glycosynthase mutants, using glycan oxazolines as substrates [22, 23]. EndoS is an endo-β-*N*-acetylglucosaminidase (ENG’ase) from *Streptococcus pyogenes* capable of hydrolyzing the Fc *N*-glycans of intact IgG antibodies by cleaving the β-1,4-glycosidic bond within the chitobiose core of *N*-glycans [24]. The glycosynthase mutants (endoS-D233A and endoS-D233Q) generated by site-directed mutagenesis have more remarkable transglycosylation activity than hydrolysis activity, such that glycosynthase mutants can transfer the complex type *N*-glycans from the corresponding oxazoline onto the GlcNAc or GlcNAcα1–6 Fuc moiety on intact antibodies [22]. Although several cell lines (yeast, CHO cells, etc.) can produce more authentic glycoproteins with non-fucosylated complex type *N*-glycans using several genetic manipulations containing glyco-related enzymes genes, glycoproteins produced from silkworm cocoon do not contain a core fucosylated glycan without gene knockdown or knockout, unlike other tissues (fat body) [25]. Their Fc *N*-glycans consist of the non-fucosylated pauci-mannose (Man_2-3_GlcNAc_2_), high-mannose (Man_4-9_GlcNAc_2_), and complex types (Man_3_GlcNAc_3-4_). In this study, we prepared homogenous mAbs with the desired *N*-glycans from anti-human epidermal growth factor receptor 2 (Her2) mAbs produced by the transgenic silkworm cocoon using a chemoenzymatic approach. EndoS cannot hydrolyze the pauci- and high-mannose types of *N*-glycans [24, 26]. Thus, we prepared endoLL, which is a novel ENG’ase from *Lactococcus lactis*. This enzyme can hydrolyze high-mannose type *N*-glycan, but not pauci-mannose or complex type *N*-glycan. Thus, we examined the activities of other ENG’ases (endoH, endoD and endoM) for IgG antibodies from silkworm cocoon. As a result, we found that *N*-glycans of the IgG antibody could be fully cleaved by the combination of endoS, endoD and endoLL. In addition, we prepared four types of complex type *N*-glycan moieties (M3, G0, G2, and A2), where G0, G2 and A2 were obtained from hen egg yolk sialylglycopeptide via enzymatic reactions [27, 28, 29], and M3 was constructed by the coupling reaction of building blocks, after which the corresponding oxazoline was synthesized using 2-chloro-1,3-dimethyl-2-imidazolinium chloride in water, as described previously [30]. Next, we performed transglycosylation of the oxazoline to GlcNAc-anti-Her2 mAbs using a glycosynthase (endoS-D233Q) as described previously [22] (Fig 1A), and obtained the glycoengineered anti-Her2 mAb with homogenous *N*-glycans (M3, G0, G2, and A2), as shown in Fig 1B. In this work, we found that the glycoengineered anti-Her2 mAbs have different affinities for the FcγRIIIa-V158 variant using the enzyme linked immunosorbent assay (ELISA) method. Next, we performed an ADCC-reporter gene assay for the glycoengineered anti-Her2 mAbs using SKBR-3 and BT-474 cells with high Her2 expression (~1 × 10^6^ molecules per cell; [31]) and Jurkat/ FcγRIIIa/NFAT-Luc cells [32].

## Materials and Methods

### Materials

The anti-Her2 mAb from CHO cells (trastuzumab) was obtained from F. Hoffmann-La Roche (Basel, Switzerland). Wild-type endoS and endoS mutant (endoS-D233Q) were overproduced in *Escherichia coli* and purified as described previously [22, 24]. Recombinant trypsin (proteomics grade) was purchased from Roche Diagnostics GmbH (Mannheim, Germany). β-1,4 Galactosidase and Remove-iT endoD were obtained from New England Biolabs (Ipswich, MA, USA). Compound 2-chloro-1,3-dimethylimidazolinium chloride (DMC) was purchased from Tokyo Chemical Industry Co., Ltd. (Tokyo, Japan). Sephadex G-15 and G-25 were purchased from GE Healthcare Life Sciences (Uppsala, Sweden). Iatrobeads 6RS-8060 were purchased from LSI Medience Corporation (Tokyo, Japan). Sepharose 4B was purchased from Sigma-Aldrich (Milwaukee, WI, USA). RapiGest SF was purchased from Waters (Milford, MA, USA). Benzoic acid *N*-succinimidyl ester was purchased from Santa Cruz Biotechnology Inc. (Dallas, TX, USA). Recombinant human FcγRIIIa-V158 was purchased from Novoprotein Scientific Inc. (Summit, NJ, USA). Microtiter plates (Nunc-Immuno, MaxiSorp surface) were purchased from Thermo Fisher Scientific Inc. (Waltham, MA, USA). Protein G-horseradish peroxidase conjugate was purchased from Bio-Rad Laboratories (Hercules, CA, USA). Compound 3, 3’,5, 5’-tetramethylbenzidine (TMB) solution was purchased from eBioscience Inc. (San Diego, CA, USA). SKBR-3 and BT-474 cells were purchased from the American Type Culture Collection (Manassas, VA, USA). The ADCC reporter bioassay kit containing Jurkat/ FcγRIIIa/NFAT-Luc cell and Bio-Glo Reagent was purchased from Promega (Madison, WI, USA). [32]. Modified amino acid analyses were performed with amino acid analyzer (Hitachi, L-8900) at the Center for Instrumental Analysis, Hokkaido University. Electrospray ionization mass spectrometry (ESI-MS) spectra were obtained with an ESI orbitrap mass spectrometer (Exactive plus EMR) equipped with Zorbax 300SB-C8 (1.0 x50 mm, Agilent Technologies Inc., Santa Clara, CA, USA) using 0.1% formic acid and acetonitrile as eluents, and deconvoluted spectra were obtained using Protein Deconvolution Software at Thermo Fisher Scientific Japan.

### Generation of transgenic silkworms carrying anti-Her2 mAb cDNAs

A DNA fragment comprised of the sericin-1 promoter and fibroin light chain polyA signal was excised from the pMSG1.1R vector [33] and inserted into the *Asc*I site of pMSG1.1MG [25]. The resulting vector, named pMSG3.1MG, carried two sericin-1 promoters and one *hr3* enhancer and was used to construct a vector for the generation of transgenic silkworms bearing anti-Her2 mAb cDNAs, as described below.

cDNAs for the mature heavy and light chains of anti-Her2 mAbs [34] were artificially synthesized. Sequences for signal peptides, sig-H (5′- ATGAACTTCGGGCTCAGCTTGATTTTCCTTGTCCTTGTTTTAAAAGGTGTCCAATGT-3′) and sig-L (5′- ATGGAGTCACAGATTCAGGCATTTGTATTCGTGTTTCTCTGGTTGTCTGGTGTTGACGGA-3′), were added to the 5′-termini of the heavy and light chain cDNAs, respectively. The 5′-UTR sequence of BmNPV polyhedrin [33] was additionally added to the 5′-termini of signal peptide-encoding sequences for both the heavy and light chains. The resulting cDNA for the heavy chain of the anti-Her2 mAb was inserted into the *Nru*I site located downstream of the sericin-1 promoter in pMSG3.1MG, followed by insertion of the light chain cDNA into the *Eco*47 III site downstream of another sericin-1 promoter within the vector. The constructed vector bearing both the heavy and light chain cDNAs was dubbed pHer2/MSG3.1.

pHer2/MSG3.1 was injected with the helper vector pHA3PIG [35] into eggs of the silkworm pnd-w1 strain, as described previously [36]. The hatched G0 larvae were reared into moths at 25°C. G1 embryos obtained by mating siblings were screened for MGFP expression in the eyes to obtain transgenic silkworms. The resultant transgenic silkworms were crossed with IM1 silkworm [37] that carried the baculovirus trans-activator IE1 to generate silkworms bearing both the antibody cDNAs and IE1 gene, which were used to produce cocoons containing recombinant anti-Her2 mAb.

### Preparation of anti-Her2 mAbs from silkworm cocoons

Silkworm cocoons containing anti-Her2 mAbs were suspended in 50 mM phosphate buffer (pH 6.7) containing 1% triton X-100 and stirred at room temperature for 1 h. The extract was filtered and applied to a SP cation-exchange column after a four-fold dilution with distilled water. The column was washed with 12.5 mM phosphate buffer (pH 6.7), and the bound proteins were eluted with 12.5 mM phosphate buffer (pH 6.7) containing 1.0 M NaCl. After buffer exchange with phosphate-buffered saline (PBS) by ultrafiltration, the antibody in the eluent was purified by protein G affinity column chromatography, as described previously [25]. The purified anti-Her2 mAb that was eluted from the column with glycine-HCl (pH 2.7) was dialyzed against PBS.

### Expression and purification of ENG’ase from *Lactococcus lactis* (endoLL)

The endoLL gene was amplified by polymerase chain reaction (PCR) from the genomic DNA of *Lactococcus lactis* subsp. lactis MAFF 516032, which was obtained from the NIAS Genebank (Tsukuba, Japan), using the upstream primer 5′- TTGGAGGATTTTATGAAAAAATCG -3′ and a downstream primer 5′- TCAGCTATTTTTTTGTCCTAATACTTG -3′. Using the amplified gene as a template, a second PCR was performed. The primers were as follows: upstream primer 5′—gggcccctgggatccAAAAAATCGAAAAAA—3′ and downstream primer 5′—atgcggccgctcgagTTAGCTATTTTTTTG—3′ (lowercase letters indicate the vector sequence for the In-Fusion cloning). The 2.8-kb amplified product was ligated into the pGEX-6P-1 *Bam*HI-*Xho*I site using the In-Fusion cloning method (Clontech Laboratories, Inc., Mountain View, CA, USA) to construct EndoLL/pGEX-6P-1. The plasmid was subsequently introduced into *E*. *coli* strain BL21 (DE3), and the transformants were induced with 50 μM IPTG for 17 h at 22°C. The pelleted cells were treated with lysozyme and sonicated, and the centrifuged supernatant was subjected to affinity chromatography on GST-Accept (Nacalai Tesque, Inc., Kyoto, Japan). EndoLL was eluted with 10 mM glutathione for the GST-tagged protein (MW 129 kDa) and recovered after Turbo3C protease digestion (Accelagen Inc., San Diego, CA, USA) for the native protein (MW 103 kDa). The buffer in the eluent was exchanged with NET buffer (50 mM Tris-HCl pH8.0, 150 mM NaCl, and 1 mM EDTA) using a Vivaspin 500 MWCO 30 K (Sartorius AG, Goettingen, Germany).

### Analysis of *N*-glycopeptides derived from anti-Her2 mAb

*N*-Glycopeptides from anti-Her2 mAbs were analyzed by MALDI-TOF MS according to a previous report [38]. Anti-Her2 mAbs (20 μg) were dissolved in 50 μL of 50 mM ammonium bicarbonate solution and heated at 100°C for 15 min. After the sample was cooled, 5 μL of 1% aqueous RapiGest SF (v/v) and 5 μL of trypsin (1.0 μg) were added, followed by incubation at 37°C for 2 days. The sample was heated at 100°C for 15 min to inactivate the enzyme, desalted by passage through a G-25 gel filtration column (0.8 × 3.5 cm), and concentrated using a centrifugal evaporation system. The samples were dissolved in 20 μL of water, 10 μL of pyridine and 20 μL of 200 mM benzoic acid *N*-succinimidyl ester solution in DMF were added and reacted at 57°C for 12 h. After addition of 60 μL of 0.5 M NaOH_aq_ to de-esterify the product, the sample was mixed on a vortex mixer at room temperature for 30 min. Water (200 μL) was added, the sample was washed with ethyl acetate (400 μL × 3 times) to remove excess reagent, and the aqueous layer was collected and concentrated using a centrifugal evaporation system. The sample was desalted on a C-18 Spin column (20 mg of C-18 reverse phase silica gel) and concentrated using a centrifugal evaporation system. Further purification was performed using the HILIC method. The sample was dissolved in 20 μL of water, added to a mixture of Sepharose 4B (wet vol. 50 μL), 100 μL of ethanol, and 400 μL of butanol and then mixed on a tube rotator at room temperature for 1 h. The resin was washed thoroughly with 2 mL of 10:2.5:2.2:0.3 (v/v/v/v) butanol/ethanol/water/formic acid to remove unglycosylated peptides, and then the labeled glycopeptides were eluted with 1.3 mL of 25% ethanol and concentrated using a centrifugal evaporation system. The sample solution (0.5~1.0 μL) was mixed with 1 μL of matrix (10 mg/mL 2,5-dihydroxybenzoic acid solution [Shimadzu Biotech, Kyoto, Japan]) on a target plate. Mass spectra were acquired in positive ion mode using a matrix-assisted laser desorption ionization quadrupole ion trap time-of-flight mass spectrometer (MALDI QIT-TOF MS) (AXIMA Resonance, Shimadzu Biotech, Manchester, UK) or a MALDI TOF MS in linear mode (AXIMA TOF^2^, Shimadzu Biotech). Ions were generated by a pulsed nitrogen UV laser (337 nm, 5 Hz).

### Cleavage of anti-Her2 mAbs from silkworm cocoon by endoS, endoM, endoD, endoLL, and endoH

The reactions of endoS, Remove-iT endoD, and endoLL (0.75 μg) were performed in 50 mM sodium phosphate, pH 7.5 containing 4 μg of anti-Her2 mAb (final volume: 20 μL) for 5 h at 37°C. Digestion by endoH and endoM (0.2 μg) were performed in 50 mM sodium citrate pH 5.5 and 50 mM sodium phosphate pH 6.0, respectively. The reaction mixture was analyzed by 10% sodium dodecyl sulfate polyacrylamide gel electrophoresis (SDS-PAGE).

### Preparation of GlcNAc-anti-Her2 mAb

A total of anti-Her2 mAb (20 mg) was digested by mixing with 2.5 μg of GST-endoS, 50 units of Remove-iT endoD, and 2.5 μg of GST-endoLL in 50 mM Tris-HCl pH 7.5 (final volume; 4 mL) for 4 h at 37°C. The reaction mixture was then subjected to affinity chromatography on GST-Accept and Chitin Resin (New England Biolabs) for removal of endoglycosidases. The unbound fraction was collected and applied to affinity chromatography on Ab-Capcher ExTra beads (ProteNova Co. Ltd., Kagawa, Japan). GlcNAc-anti-Her2 mAbs were eluted with 100 mM glycine-HCl pH 2.8 and the buffer in the eluent was exchanged with PBS using a Vivaspin 500 MWCO 50 K (Sartorius AG, Goettingen, Germany).

### Purity analysis of anti-Her2 mAbs by cation-exchange HPLC

Cation-exchange HPLC analysis was performed on the Shimadzu HPLC system equipped with UV detection (280 nm) using a ProPac WCX-10 (4 × 250 mm, Thermo Fisher Scientific Inc., Sunnyvale, CA, USA) and a gradient elution (phase A: 10 mM sodium acetate (pH 4.15); phase B: 10 mM sodium acetate (pH 4.15) + 1.0 M NaCl), as described previously [39, 40]. The sample was eluted with gradients of 0% NaCl (0–5 min), 0–100% NaCl (5–105 min), washed with 100% NaCl (105–120 min), and equilibrated with 0% NaCl (120–140 min) at a constant flow of 1.0 mL/min (column oven at 30°C).

### Preparation of oligosaccharide oxazolines (M3-Oxa, G0-Oxa, G2-Oxa, and A2-Oxa)

GlcNAc_2_Man_3_GlcNAc_1_ (G0-OH), Gal_2_GlcNAc_2_Man_3_GlcNAc_1_ (G2-OH), and NeuAc_2_Gal_2_- GlcNAc_2_Man_3_GlcNAc_1_ (A2-OH) were prepared from hen egg yolk sialylglycopeptide (SGP) consisting of disialylated biantennary *N*-glycan (A2) with a specific amino acid sequence (KVA*N*KT), as described previously [27, 41] with minor modifications. Briefly, SGP was hydrolyzed by 12.5 mM HCl at 95°C for 2.5 h to remove sialic acid residues. The resulting products were neutralized with 100 mM NaHCO_3_ and then subjected to a Sephadex G-25 gel filtration column equilibrated with 5 mM NH_4_HCO_3_ to obtain glycopeptides containing asialo-biantennary *N*-glycan (G2). This *N*-glycan was digested with β1,4-galactosidase at 37°C for 48 h in 50 mM sodium citrate buffer containing 100 mM NaCl (pH 6.0). The reaction mixture was subjected to a Sephadex G-25 gel filtration column equilibrated with 5 mM NH_4_HCO_3_ to obtain glycopeptides containing agalacto-biantennary *N*-glycan (G0). These glycopeptides with G0, G2, and A2 were then subjected to hydrolysis with wild-type endoS at 37°C for 48 h in PBS buffer (pH 7.0). G0-, G2-, and A2-OH were obtained after purification using Iatrobead column chromatography (ethly acetate/methanol/water = 100:50:2 (v/v/v) as eluent) and Sephadex G-25 gel filtration equilibrated with 5 mM NH_4_HCO_3_. Man_3_GlcNAc_1_ (M3-OH) was prepared by chemical synthesis, as described previously [42]. M3-, G0-, G2-, and A2-OH were converted into oxazolines following the previous method [30] using DMC in the presence of triethylamine. Briefly, M3-, G0-, G2-, and A2-OH were treated with DMC (30 mol equiv.) in the presence of trimethylamine (75 mol equiv) at room temperature for 4 h. M3 oxazoline, G0 oxazoline, G2 oxazoline, and A2 oxazoline (M3-Oxa, G0-Oxa, G2-Oxa, and A2-Oxa) were then purified using a Sephadex G-15 gel filtration column equilibrated with 0.05% triethylamine and lyophilized.

### Transglycosylation of GlcNAc- anti-Her2 mAbs with M3-Oxa, G0-Oxa, G2-Oxa, and A2-Oxa by endoS-D233Q

GlcNAc-anti-Her2 mAbs (2 mg) were mixed with 1.875 μmol of oxazolines (donor / acceptor molar ratio of 150:1) and 200 μg of GST-tagged endoS-D233Q in 50 mM Tris-HCl (500 μL) for 3 h at 37°C. The reaction mixture was applied to affinity chromatography on GST-Accept to remove GST-tagged endoS-D233Q. The unbound fraction was mixed with Ab-Capcher ExTra beads and gently shaken at room temperature for 1 h to capture transglycosylation products. The products were eluted with 100 mM glycine-HCl pH 2.8 and exchanged with PBS using a Vivaspin 500 MWCO 50 K (Sartorius AG, Goettingen, Germany) and analyzed by 10% SDS-PAGE.

### Isolation of fully glycosylated anti-Her2 mAbs with homogenous *N*-glycans

Glycoengineered anti-Her2 mAbs were separated using an ÄKTA FPLC system (GE Healthcare, Piscataway, NJ, USA) at 4°C. The prepared glycoengineered anti-Her2 mAbs (approximately 300 μg) were loaded onto a Mono S column (4.6 × 100 mm, GE Healthcare) and separated using the following stepwise gradient (phase A: 25 mM sodium acetate (pH 4.15); phase B: 25 mM sodium acetate (pH 4.15) + 500 mM NaCl) at a constant flow of 1.35 mL/min. For A2-anti-Her2 mAb (mAb-A2), 50 mM NaCl (10 CV), 50–275 mM (10 CV), 275–300 mM (10 CV), 300–325 mM (12.5 CV), 325 mM (12.5 CV), 325–350 mM (12.5 CV), 350 mM (12.5 CV), 350–400 mM (10 CV), 400–500 mM (5 CV), and 500 mM (10 CV). For G2-anti-Her2 mAb (mAb-G2), 50 mM NaCl (10 CV), 50–275 mM (10 CV), 275–325 mM (10 CV), 325 mM (10 CV), 325–340 mM (10 CV), 340 mM (15 CV), 340–365 mM (12.5 CV), 365 mM (12.5 CV), 365–390 mM (10 CV), 390–500 mM (5 CV), and 500 mM (10 CV). For G0-anti-Her2 mAb (mAb-G0), 50 mM NaCl (10 CV), 50–275 mM (10 CV), 275–330 mM (10 CV), 330 mM (12.5 CV), 330–345 mM (10 CV), 345 mM (15 CV), 345–370 mM (12.5 CV), 370 mM (12.5 CV), 370–395 mM (10 CV), 395–500 mM (5 CV), and 500 mM (10 CV). For M3-anti-Her2 mAb (mAb-M3), 50 mM NaCl (10 CV), 50–275 mM (10 CV), 275–350 mM (15 CV), 350 mM (15 CV), 350–365 mM (10 CV), 365 mM (15 CV), 365–390 mM (12.5 CV), 390 mM (12.5 CV), 390–415 mM (10 CV), 415–500 mM (5 CV), and 500 mM (10 CV). Each fraction contained 2.0 mL/tube. The purified glycoengineered anti-Her2 mAbs were collected and concentrated using an Amicon Ultra-15 centrifugal unit (EMD Millipore, Billerica, MA, USA) in 25 mM Tris-HCl buffer (pH 8.0); the purification and yield were confirmed by cation-exchange HPLC analysis.

### Protein concentration

The concentration of glycoengineered anti-Her2 mAbs for FcγRIIIa binding assays was determined using the human IgG ELISA quantitation kit (Bethyl Laboratories Inc., Montgomery, TX, USA).

### FcγRIIIa binding assay

The binding affinity of each purified mAb for recombinant human FcγRIIIa-V158 was measured using an FcγRIIIa binding ELISA, as described previously [43]. A FcγRIIIa-V158 solution in PBS (100 μl, 10 μg/mL) was coated onto microtiter plate wells (Nunc-Immuno, MaxiSorp surface, Thermo Fisher Scientific Inc., Waltham, MA, USA) overnight at 4°C. The wells were washed five times with 200 μl of tris-buffered saline with tween 20 (pH 8.0) between each step to thoroughly remove unbound material. The wells were blocked with 200 μl of tris-buffered saline containing 1.0% (w/v) bovine serum albumin (pH 8.0) at 25°C for 1 h. The purified glycoengineered anti-Her2 mAbs (100 μl, 9.0–0.0041 μg/mL) were added to wells and incubated for 2 h. After washing, bound antibodies to FcγRIIIa-V158 were detected using HRP-conjugated protein G. The substrate used was TMB solution (100 μl). After addition of 0.18 M H_2_SO_4_ (100 μl), the absorbance at 450 nm was read using a V_max_ plate reader (Molecular Devices, Sunnyvale, CA, USA). All samples were assayed in duplicate. All data were plotted using GraphPad Prism 6 software (GraphPad Software, Inc., La Jolla, CA, USA).

### FcγR reporter assay

The assay was performed as a luciferase assay using ADCC reporter cells that have Fcγ receptors and the response element-driven luciferase gene (Promega, ADCC Reporter Bioassay Complete Kit (Raji)) [32]. The Her2-positive human breast carcinoma cell lines, SKBR-3 and BT-474, were used as target cells and adapted to RPMI 1640 medium supplemented with 10% fetal bovine serum (FBS). The day before the assay, target cells were seeded at 1,500 cells/well in 96-well tissue culture plates in ADCC assay medium (RPMI 1640 medium supplemented with MEM non-essential amino acids and 4% super-low IgG FBS) (HyClone Laboratories, Logan, UT, USA). The next day, anti-Her2 mAbs serially diluted in the ADCC assay medium were incubated with target cells for approximately 30 minutes at 37°C. Following incubation, Jurkat ADCC reporter cells suspended in ADCC assay medium were added to the target cell/antibody mixture at 75,000 cells/well. After approximately a 20-h incubation at 37°C, 5% CO_2_, an equal volume of the Bio-Glo Luciferase assay reagent (Promega) was added to the wells. After a 15-minute incubation at room temperature, 30 seconds of integrated luminescence were measured using a Lumat LB 96V (Berthold Technologies GmbH & Co. KG, Bad Wildbad, Germany). Assays were performed in triplicate. All data were plotted using GraphPad Prism 6 software.

## Results

### Production of anti-Her2 mAbs from silkworm cocoon

**Fig 2:**
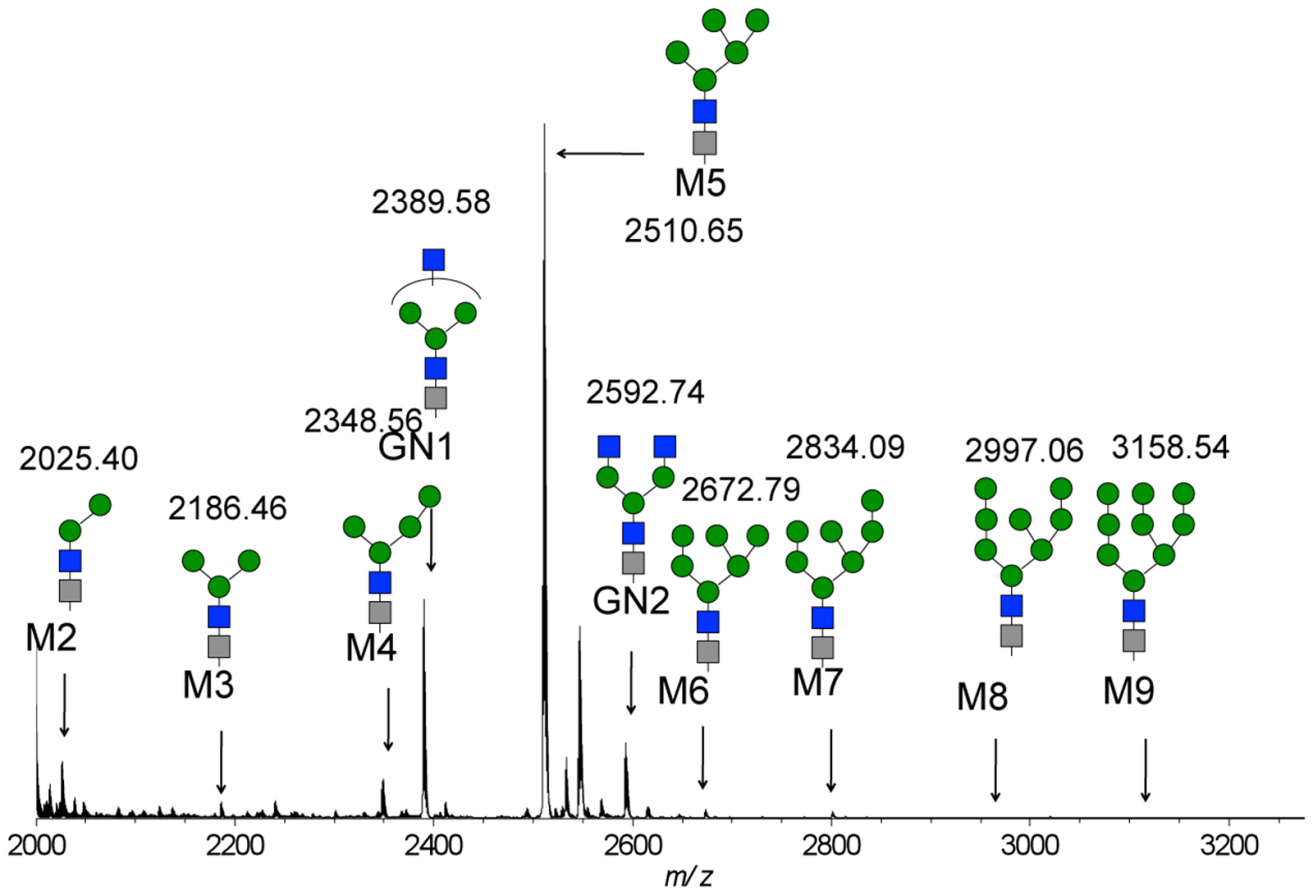
MALDI QIT-TOF MS spectrum of Bz-labeled glycopeptides from anti-Her2 mAbs produced in silkworm cocoon.

The pHer2/MSG3.1 vector was constructed and injected the vector into silkworm eggs. Hatched larvae were allowed to develop to moths, and embryos obtained by mating the moths were screened for MGFP expression to obtain transgenic silkworms carrying anti-Her2 mAb cDNAs. The resultant transgenic silkworms were crossed with IM1 silkworms carrying the baculovirus trans-activator IE1 to generate silkworms bearing both the mAb cDNAs and IE1 gene, which were used to produce cocoons containing recombinant anti-Her2 mAb. A transgenic cocoon extract prepared with 50 mM phosphate buffer (pH 6.7) containing 1% triton X-100 was applied to a SP cation-exchange column. Bound proteins were eluted with 12.5 mM phosphate buffer (pH 6.7) containing 1.0 M NaCl, and the buffer in the eluent was exchanged with PBS. The anti-Her2 mAb was then purified by protein G affinity column chromatography, as described previously [25]. We obtained 397.9 mg of purified anti-Her2 mAb from 125 g of transgenic cocoons. Anti-Her2 mAbs from silkworm cocoon have no core fucosylated glycan, unlike other tissues (fat body) [25]. We observed Bz-labeled glycopeptides consisting of nine amino acids (EEQYNSTYR) via tryptic digestion, which were derived from the constant region of the heavy chains of anti-Her2 mAbs and reacted with BzOSu to increase peptide mass by 104 Da and enhance the detection sensitivity of MALDI-TOF MS according to previous methods [38]. In Fig 2, the glycopeptides consisting of at least 10 glycan compositions (Hex_2_HexNAc_2_ (M2), Hex_3_HexNAc_2_ (M3), Hex_4_HexNAc_2_ (M4), Hex_3_HexNAc_3_ (GN1), Hex_5_HexNAc_2_ (M5), Hex_3_HexNAc_4_ (GN2), Hex_6_HexNAc_2_ (M6), Hex_7_HexNAc_2_ (M7), Hex_8_HexNAc_2_ (M8), and Hex_9_HexNAc_2_ (M9)) and the same amino acid sequence (EEQYNSTYR), which were assigned from MS1 and major peaks were characterized with MS/MS analysis, were observed at *m/z* 2025.40 (5.04%, calcd *m/z* 2023.80), 2186.46 (1.48%, calcd *m/z* 2185.86), 2348.56 (3.55%, calcd *m/z* 2347.91), 2389.58 (19.74%, calcd *m/z* 2388.94), 2510.65 (62.57%, calcd *m/z* 2509.96), 2592.74 (6.75%, calcd *m/z* 2592.01), 2672.79 (0.73%, calcd *m/z* 2672.01), 2834.09 (0.09%, calcd *m/z* 2834.07), 2997.06 (0.03%, calcd *m/z* 2996.12), and 3158.54 (0.01%, calcd *m/z* 3158.17), respectively (% is calculated from total 10 glycopeptides average peak intensities in triplicate, calcd *m/z* is theoretical value of [M+H]^+^.). From these data, we found that the Fc *N*-glycans produced in the silkworm cocoon consisted of the non-fucosylated pauci-mannose (Man_2-3_GlcNAc_2_), high-mannose (Man_4-9_GlcNAc_2_), and complex types (Man_3_GlcNAc_3-4_). Next, we showed that anti-Her2 mAbs from silkworm cocoon consist of 72.7% fully glycosylated mAb, 23.4% hemi-glycosylated mAb, and 3.8% aglycosylated mAb based on cation-exchange HPLC analysis [30, 40] (S1 Fig), and the ratio of antibody glycosylation was 88.5%, based on the calculated amount of GlcNAc residues, using the modified amino acid analysis compared to anti-Her2 mAb from CHO cells as 100% glycosylation ratio. Thus, we developed a cation-exchange chromatography (IEX column) method for the separation of fully glycosylated and hemi-glycosylated intact antibodies (S2 Fig) and confirmed the glycan distribution and combination of intact antibodies before and after separation using ESI-MS analysis (S3 Fig). Although the micro-heterogeneity of *N*-glycans on the Fc domain of the heavy chain consists of 10 glycoforms, the heterogeneity of the intact antibody, which influences the combination and distribution of glycoforms, consisted of more than 10 forms (GN1+M3, GN1+GN1 (or M3+GN2), M4+M5, GN1+M5, GN2+M4, GN1+GN2, M5+M5 (or M4+M6), GN1+M6, GN2+M5, and M5+M6 (M4+M7)) based on the deconvoluted spectrum (S3 Fig). The heterogeneity of intact antibodies containing M2, M8 and M9 was barely detected due to contents that were too low.

### Preparation of carbohydrate-hydrolyzed anti-Her2 mAb

**Fig 3:**
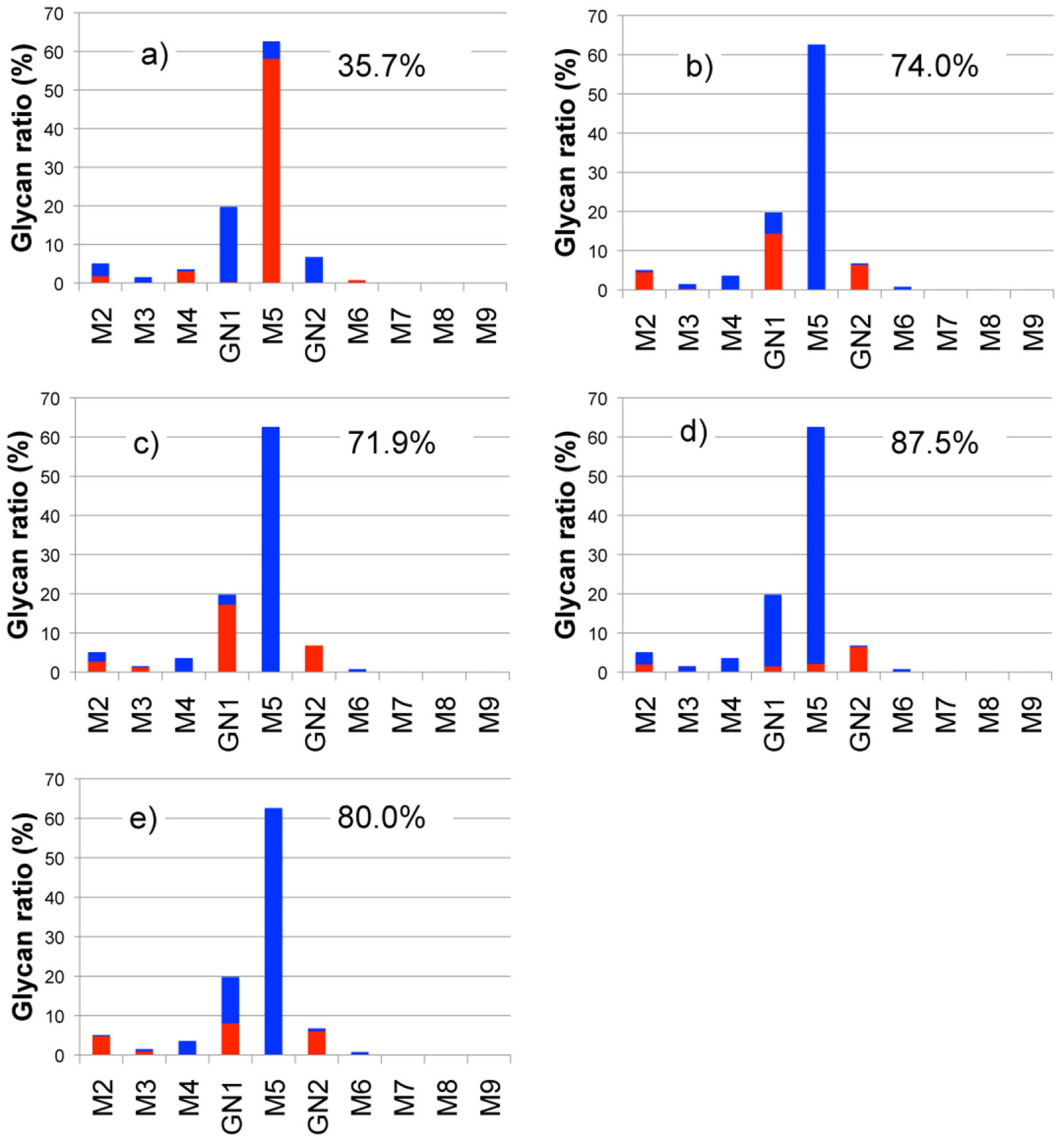
ENG’ase activity of the anti-Her2 mAbs (a; endoS, b; endoD, c; endoH, d; endoM, e; endoLL). (Blue bar represents glycopeptides without ENG’ase hydrolysis; red bar represents the remaining glycopeptides with ENG’ase hydrolysis; y-axis indicates each individual glycoform ratio to total glycoform content; % represents total cleaved glycopeptide ratio by ENG’ase hydrolysis.)

For efficient chemoenzymatic synthesis of glycoengineered mAb, it is important to characterize the substrate specificities of endo-ß-*N*-acetylglucosaminidases (ENG’ases). Thus, we analyzed ENG’ase activity for anti-Her2 mAbs using MALDI-TOF MS, in which the hydrolyzed glycoform could be estimated based on comparative analysis of relative intensities of mAb glycopeptides with/without the ENG’ase reaction (endoH, endoD, endoM, endoS, and endoLL, S1 Text.) from MS spectra, as shown in Fig 3a–3e, S4 and S5 Figs. ENG’ases are a class of enzymes (glycoside hydrolase families 18 and 85) that cleave the chitobiose core (GlcNAc-ß-1,4-GlcNAc) of *N*-glycans. In this analysis, the glycoforms on the mAbs, representing a product of the ENG’ase reaction, could be detected directly. Moreover, the decreased amount of glycopeptides hydrolyzed by ENG’ase could be estimated by comparative analysis of glycopeptides with and without ENG’ase hydrolysis. Various ENG’ases have been identified from numerous natural sources (bacteria, plants, and animals) [44, 45, 46]. ENG’ase from *Streptomyces plicatus* (endoH) is active on high-mannose type *N*-glycans [47], and was able to completely hydrolyze M4~9 glycoforms on mAbs (Fig 3c). ENG’ase from *Streptococcus pneumoniae* (endoD) cleaves only *N*-glycans with an unsubstituted α-mannosyl residue at the C-3 position of the terminal mannose of the *N*-glycan core structure from glycopeptides [48], and was able to cleave M3~5 and a portion of GN1 (Fig 3b). ENG’ase from *Mucor hiemalis* (endoM), which hydrolyzes non-fucosylated *N*-glycans containing high-mannose and complex types [49], was able to remove the majority of *N*-glycans, excluding GN2 (Fig 3d). ENG’ase from *Streptococcus pyogenes* (EndoS) has the ability to hydrolyze non-fucosylated and fucosylated *N*-glycans on the Fc domain of intact IgG antibodies [24], but was not capable of hydrolyzing the pauci- and high-mannose type *N*-glycans (Fig 3a). To fully hydrolyze the non-fucosylated pauci-mannose, high-mannose, and complex types on intact mAbs from silkworm cocoon, we searched for novel ENG’ases from *Lactococcus lactis* (endoLL), which has similar amino acid sequences to those of other ENG’ases (25% identity with endoM, 29% identity with endoD, 0% identity with endoH, 0% identity with endoF1). EndoLL can hydrolyze high-mannose type *N*-glycans (M4~9) and a portion of GN1, but not pauci-mannose or complex type *N*-glycans (Fig 3e). Since one type of ENG’ase could not completely hydrolyze *N*-glycans on mAbs, we performed the hydrolysis of mAb *N*-glycan using a combination of endoS, endoD and endoLL to be active towards all glycans under suitable conditions (pH 7.5) with three types of ENG’ase activities (S6 Fig). As a result, we prepared fully carbohydrate-hydrolyzed anti-Her2 mAbs containing monosaccharides (GlcNAc) on the Fc domain (GlcNAc-anti-Her2 mAb).

### Preparation of homogeneous glycosylated (M3, G0, G2, and A2) anti-Her2 mAbs

**Fig 4:**
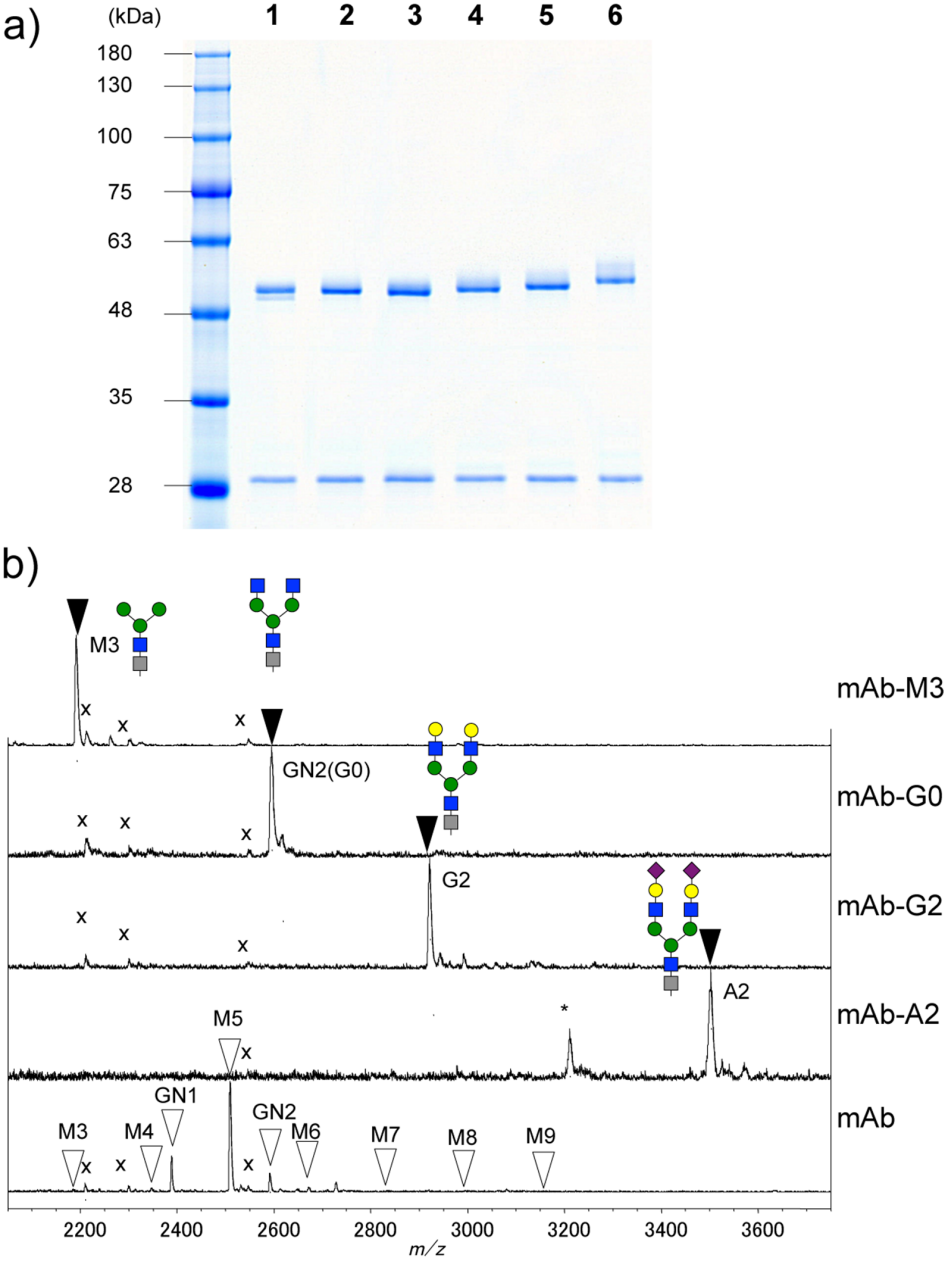
a) SDS-PAGE analysis of lane 1; anti-Her2 mAb from silkworm cocoon before IEX column chromatography (52.5 and 53.7 kDa as heavy chains (H), 29.8 kDa as light chains (L)), lane 2; anti-Her2 mAb from silkworm cocoon after IEX column chromatography (mAb; 53.7 kDa (H), 29.8 kDa (L)), the homogeneous glycosylated anti-Her2 mAb with M3 after IEX column (mAb-M3, lane 3; 53.5 kDa (H), 29.8 kDa (L)), G0 after IEX column chromatography (mAb-G0, lane 4; 53.7 kDa (H), 29.8 kDa (L)), G2 after IEX column chromatography (mAb-G2, lane 5; 54.1 kDa (H), 29.8 kDa (L)), and A2 after IEX column chromatography (mAb-A2, lane 6; 55.1 kDa (H), 29.8 kDa (L)). MW was calculated from the band mobilities with regards to molecular markers. b) MALDI-TOF MS spectra of glycopeptides from mAb-M3, mAb-G0, mAb-G2, mAb-A2, and mAb in the positive mode. * indicates the de-sialylated fragment ion peak. x represents the common contaminant peaks, not glycopeptides.

Glycoengineered anti-Her2 mAbs with homogenous *N*-glycans (M3, G0, G2, and A2) were prepared using a chemoenzymatic approach, which transglycosylated an oxazoline to GlcNAc-anti-Her2 mAb using a glycosynthase (endoS-D233Q), as described previously [22]. Three types of *N*-glycan moieties consisting of GlcNAc_2_Man_3_GlcNAc_1_ (G0-OH), Gal_2_GlcNAc_2_Man_3_GlcNAc_1_ (G2-OH) and NeuAc_2_Gal_2_GlcNAc_2_Man_3_GlcNAc_1_ (A2-OH) were prepared from hen egg yolk SGP via a continuous hydrolysis reaction by β-galactosidase (for G0), acid-hydrolysis (for G2 and G0) and endoS (for G0, G2 and A2) [27, 41], and were confirmed using ^1^H NMR analysis (S7, S8 and S9 Figs) [41, 50, 51]. Although endoS is known to have high selective activity for *N*-glycans on the Fc domain of IgG [22], endoS can also cleave a series of *N*-glycans conjugated to a peptide [29] [38]. Man_3_GlcNAc_1_ (M3-OH) was prepared by chemical synthesis using a carbohydrate building block according to a previous report [42] and identified using NMR analysis (JEOL, JNM-ECA600, 600 MHz) [52] (S10 Fig). Shoda *et al*. reported an efficient method for the preparation of oligosaccharide oxazolines from free oligosaccharides in aqueous solution using DMC as the reagent for a single-step conversion [30]. Thus, we followed this procedure to prepare M3-Oxa, G0-Oxa, G2-Oxa, and A2-Oxa, which were obtained at excellent yields. M3-Oxa, G0-Oxa, G2-Oxa, and A2-Oxa were identified using ^1^H NMR analysis, which showed the characteristic signal of the GlcNAc proton in an oxazoline form as a doublet peak with a specific coupling constant (M3-Oxa; 6.12 ppm, 6.87 Hz, G0-Oxa; 6.14 ppm, 6.87 Hz, G2-Oxa; 6.11 ppm, 7.56 Hz, A2-Oxa; 6.13 ppm, 7.56 Hz) (S11, S12, S13 and S14 Figs). These oxazolines were immediately used for the preparation of glycoengineered mAbs without further purification, because they are easily decomposed under acidic and neutral conditions (S15 Fig). Although glycosylation remodeling of glycoproteins by chemoenzymatic approaches using endoD and endoA mutants has been reported [53, 54], we applied a chemoenzymatic approach using an endoS mutant, which has high-reactivity for intact full-length IgG antibodies based on a report by Wang *et al*. [22]. First, we monitored the transglycosylation reaction using G2-Oxa as a donor substrate and GlcNAc-anti-Her2 mAbs as an acceptor using the endoS-D233Q mutant for 0–6 h based on SDS-PAGE analysis (S16 Fig). By monitoring the reaction, the transglycosylation product showed the best yield after 3 h because it preceded hydrolysis by the endoS mutant. We performed transglycosylation of the oxazoline to GlcNAc-anti-Her2 mAbs using endoS-D233Q. The reaction mixture was quickly isolated by IEX column chromatography to prevent product hydrolysis, and the separation of glycoengineered mAbs between fully glycosylated and hemi-glycosylated mAbs was confirmed by SDS-PAGE (Fig 4a, S17 Fig) and cation-exchange HPLC analysis (S18, S19, S20 and S21 Figs). Fully glycosylated mAbs consist of two completely reacted glycosylated heavy chains and two light chains, whereas hemi-glycosylated mAbs consist of a single reacted glycosylated heavy chain and a single unreacted aglycosylated heavy chain and two light chains. We obtained fully glycosylated mAbs using IEX column chromatography since unreacted aglycosylated heavy chains produced from hemi-glycosylated mAbs were not detected by SDS-PAGE (Fig 4a). Based on MALDI-TOF MS analysis in linear mode of *N*-glycopeptides prepared from glycoengineered anti-Her2 mAbs (Fig 4b), we observed Bz-labeled glycopeptides consisting of Hex_3_HexNAc_2_ (M3), Hex_3_HexNAc_4_ (G0), Hex_5_HexNAc_4_ (G2), and NeuAc_2_Hex_5_HexNAc_4_ (A2), and the same amino acid sequence (EEQYNSTYR) as *m/z* 2186.0 (calcd *m/z* 2185.9), 2592.0 (calcd *m/z* 2592.0), 2916.0 (calcd *m/z* 2916.1), and 3498.8 (calcd *m/z* 3498.3), respectively. Here, we found that the homogeneous glycosylated anti-Her2 mAb (mAb-M3, mAb-G0, mAb-G2, and mAb-A2) had homogeneous glycosylated *N*-glycan on the Fc domain of full-length anti-Her2 mAb. In the case of mAb-A2 (Fig 4b), the de-sialylated fragment ion partially appeared as *m/z* 3207.5 because the loss of sialic acid occurs due to post-source decay during MALDI-TOF MS measurement in positive mode. As a result, we performed remodeling of homogeneous *N*-glycan from heterogeneous *N*-glycan on anti-Her2 mAbs and obtained fully glycosylated mAbs containing a designated glycan.

### Effects of glycan structure on anti-Her2 mAbs for FcγRIIIa binding activity

**Fig 5:**
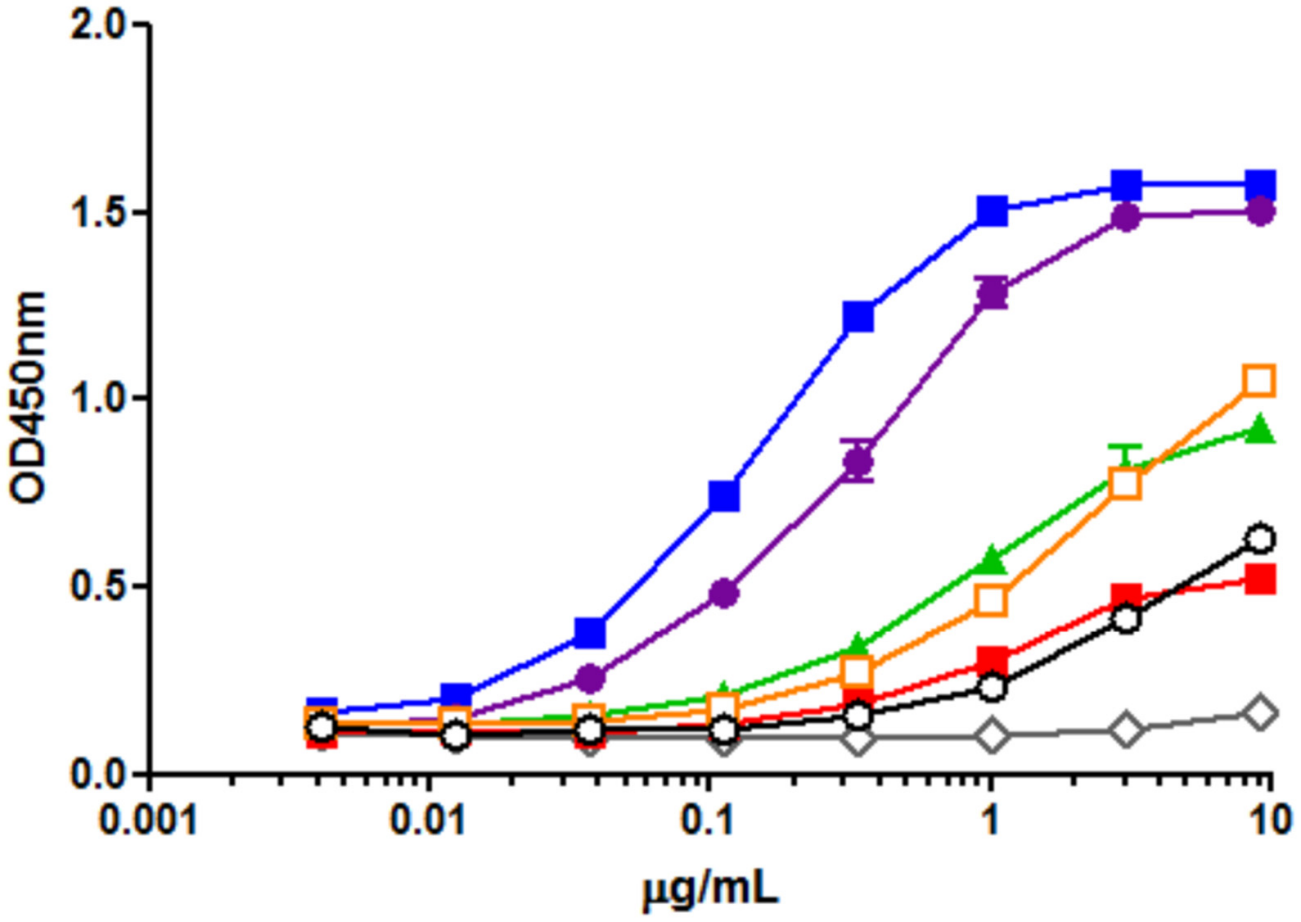
Binding activity for FcγRIIIa of the glycoengineered anti-Her2 mAbs (mAb-M3; red square, mAb-G0; green triangle, mAb-G2; blue square, mAb-A2; purple circle), aglycosylated anti-Her2 mAb (mAb-PNGF; open diamond), fully glycosylated anti-Her2 mAb from silkworm cocoon (mAb; open square), and anti-Her2 mAb from CHO cells (trastuzumab; open circle) using the FcγRIIIa-V158-binding ELISA method.

FcγRIIIa (FcγIIIa receptor), expressed on natural killer cells and macrophages through its binding activity with the Fc domain of mAbs, is mainly responsible for ADCC of therapeutic mAbs [55]. It has been reported that the presence of *N*-glycans linked to Asn-297 of the IgG1-Fc domain are important for the binding of Fc to FcγRIIIa [4], and the removal of the core fucose from Fc *N*-glycans significantly enhances the binding activity for FcγRIIIa [15, 16, 56, 57, 58, 59]. It has also been shown that hemi-glycosylated mAbs significantly reduce FcγR binding affinity compared with fully glycosylated mAbs [40]; thus, we analyzed the affinity of FcγRIIIa after the isolation of fully glycosylated mAbs to eliminate mixtures of hemi-glycosylated and aglycosylated mAbs. We examined the affinity of glycoengineered anti-Her2 mAbs (mAb-M3, mAb-G0, mAb-G2, and mAb-A2), aglycosylated anti-Her2 mAbs (mAb-PNGF), which are cleaved by PNGase F digestion, fully glycosylated anti-Her2 mAbs from silkworm cocoon (mAb), and anti-Her2 mAbs from CHO cells (trastuzumab), using a FcγRIIIa-V158-binding ELISA after measuring their concentrations using a human IgG ELISA quantitation kit, as described previously [43]. As shown in Fig 5, FcγRIIIa-V158 binding assays showed that the binding affinity depended on the glycan structures. The binding affinities (in decreasing order) at lower concentrations (less than 0.3 μg/ml) were as follows: mAb-G2 > mAb-A2 >> mAb-G0, mAb > mAb-M3, trastuzumab >> mAb-PNGF. By comparing mAb-G2 and mAb-A2, we observed decreasing affinities with sialylation that agreed with previous reports at lower concentrations [60, 61]. Although anti-Her2 mAbs from CHO cells (trastuzumab) mainly consist of fucosylated complex type *N*-glycans (S22 Fig), the heterogeneous *N*-glycans on trastuzumab were similar to the homogeneous M3-mAb in terms of the affinity for FcγRIIIa-V158. In addition, heterogeneous *N*-glycans on mAbs, on which the majority of *N*-glycans was M5 (approximately 63%), had the same affinity as homogeneous G0-mAb. These relationships between glycan structure and affinity for FcγRIIIa-V158 were clearer than those in a previous report using humanized IgG in glycoengineered *Pichia pastoris* [18], owing to the purification of fully glycosylated mAbs and the construction of homogeneous glycosylated mAbs using a chemoenzymatic approach.

### Influence of glycan structure on anti-Her2 mAbs in ADCC-reporter gene assays

**Fig 6:**
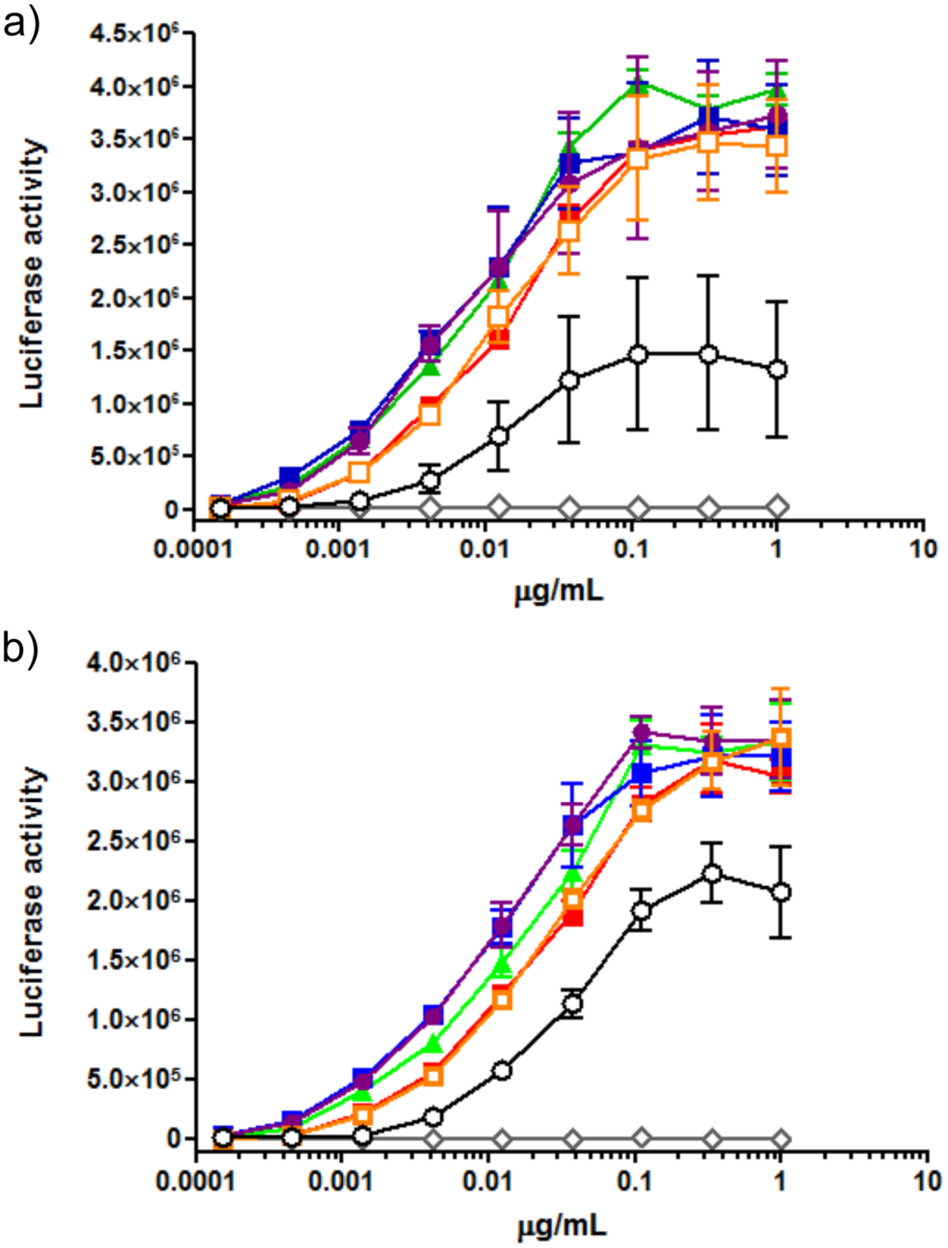
ADCC reporter gene assay of the glycoengineered anti-Her2 mAbs (mAb-M3; red square, mAb-G0; green triangle, mAb-G2; blue square, mAb-A2; purple circle), aglycosylated anti-Her2 mAb (mAb-PNGF; open diamond), fully glycosylated anti-Her2 mAb from silkworm cocoon (mAb; open square), and anti-Her2 mAb from CHO cells (trastuzumab; open circle) in SKBR-3 (a) and BT474 (b) target cells.

ADCC is a mechanism of cell-mediated immune defense whereby effector cells of the immune system actively lyse target cells, whose membrane-surface antigens are bound by specific antibodies. Thus, ADCC is an important function of therapeutic monoclonal antibodies against target cells. Recently, ADCC reporter gene assays have been developed as an alternative method without the isolation of peripheral blood mononuclear cells (PBMCs) from fresh blood [32]. This assay was performed using a recombinant Jurkat T cell line that stably expresses the FcγRIIIa complex and the luciferase reporter gene under control of the nuclear factor of activated T cell (NFAT) response elements from the IL-2 promoter, instead of PBMCs as the effector cells. The ADCC reporter gene assay correlates with PBMC-based ADCC assays and luciferase reactivity of represents ADCC activity [32, 62]. Generally, ADCC assays depend on the NK cell population of PBMCs, which induces cell killing and the expression of activated FcγRIIIa [32, 40, 63]. Thus, we performed ADCC reporter gene assays for the glycoengineered anti-Her2 (mAb-M3, mAb-G0, mAb-G2, and mAb-A2), aglycosylated anti-Her2 (mAb-PNGF), and fully glycosylated anti-Her2 mAbs from silkworm cocoon (mAb), and anti-Her2 mAbs from CHO cells (trastuzumab) to prevent the dispersion of ADCC activity derived from the preparation of PBMCs. Two breast cancer cell lines with high Her2 expression were chosen as the target cells: SKBR-3, high Her2 expression (9.76 × 10^5^ molecules per cell; and BT-474, high Her2 expression, 6.91 × 10^5^ molecules per cell [31]. The ADCC reporter assay was performed by adding effector cells, target cells (E:T ratio, 50:1), and each anti-Her2 mAb at various concentrations. It was found that the *N*-glycan structure on mAbs does not affect Her2 binding (K_D_; 0.13 and 0.12 nM for trastuzumab and afucosylated trastuzumab, respectively) [60, 63]. As shown in Fig 6a and 6b, ADCC reporter gene assays showed that ADCC activity depends on glycan structure. It was previously reported that the lack of the core fucose from Fc *N*-glycans significantly enhances ADCC activity compared to defucosylated and conventional mAbs [15], and we observed this phenomenon for both SKBR-3 and BT-474 cells, excluding aglycosylated mAbs (mAb-PNGF). The ADCC activities (in decreasing order) at lower concentrations (less than 0.01 μg/ml) were as follows: mAb-A2, mAb-G2, mAb-G0 > mAb, mAb-M3 > trastuzumab >> mAb-PNGF, in the case of SKBR-3 cells (Fig 6a), and mAb-A2, mAb-G2 > mAb-G0 > mAb, mAb-M3 >> trastuzumab >>> mAb-PNGF, in the case of BT-474 cells (Fig 6b). In both cases, the activity of the complex types (mAb-A2, mAb-G2and mAb-G0) was stronger than that of the high-mannose types (mAb and mAb-M3) at the lower concentrations (0.0001–0.1 μg/mL). Although trastuzumab and mAb-M3 have similar affinities for FcγRIIIa on effector cells (Fig 5), their ADCC activities were quite different (Fig 6). This may reflect the activity of IgG in the classical pathway of complement activation on target cells in the Her2-binding process of mAbs at low concentrations (approximately 0.0195 μg/mL), which is initiated by the IgG Fc region binding to C1q (complement component 1q) [15, 17] and mannose-binding protein [64, 65], which structurally resembles C1q, and not the affinity of FcγRIIIa on effector cells at the middle concentration range (0.01–1.0 μg/mL). The terminal Gal residue is known to enhance CDC activity by increasing the binding of antibodies to C1q [15]. ADCC activity of mAb-G2 was higher than that of mAb-G0 and mAb-M3 in the case of BT-474 cells, but Prang *et al*. reported that CDC activity of trastuzumab on several breast cancer cell lines (SKBR-3, BT-474, etc) could not be observed at concentrations up to 50 μg/mL [31]. Moreover, in the case of SKBR-3, the ADCC activities of mAb-A2, mAb-G2 and mAb-G0 were similar. Thus, we think that the differences in ADCC responses of the homogeneous glycoengineered mAb between SKBR-3 and BT-474 are suggestive of other functions.

## Discussion

We prepared glycoengineered anti-Her2 mAbs (mAb-M3, mAb-G0, mAb-G2, and mAb-A2) from anti-Her2 mAbs produced in transgenic silkworm cocoon according to a chemoenzymatic procedure, as described in Fig 1A. Glycoproteins from transgenic cocoon extracts consist of non-fucosylated *N*-glycan [26] and are suitable for the preparation of mAbs without a core fucose, which will have higher ADCC activity than that of conventional mAbs with a core fucose [15, 16, 56, 57, 58, 59]; thus, this was chosen as the starting material. Wang *et al*. prepared a glycoengineered anti-CD20 antibody without a core fucose using a chemoenzymatic approach including treatment with α-fucosidase for 3 weeks from an anti-CD20 antibody produced in CHO cells (rituximab) [22]. Owing to advances in genetic engineering, several transgenic cell lines have been developed to produce therapeutic mAbs with a depleted core fucose or galactosylated and sialylated extensions [14–20]. However, a few non-human glycan epitopes (Galα1-3Gal, NeuGc, etc) produced from transgenic cell lines are known to affect the immunogenic response, including the risk of anaphylaxis [66], and it is important to control *N*-glycans in the biosynthetic pathway. Recently, mogamulizumab (anti-CCR4 antibody, Kyowa Hakko Kirin) and obinutuzumab (anti-CD20 antibody, Genentech/Roche) with depleted core fucoses have been approved for marketing, and several glycoengineered antibodies are in clinical studies and are being developed as next generation therapeutic monoclonal antibodies. Generally, these mAbs consist of partially controlled *N*-glycans by a few gene knockouts or knockdowns and are heterogeneous glycoproteins with a broad distribution and combination of *N*-glycans [14, 18, 19]. We developed a highly sensitive analysis for glycan profiling of glycoproteins, which can be detected until the glycan content reaches less than 1% (Fig 2) [38]. We also prepared homogeneous GlcNAc-anti-Her2 mAbs as glycosyl acceptors of transglycosylation reactions using hydrolysis combined with endoS, endoD, and endoLL, which could cleave all *N*-glycans on mAbs until glycan levels reached less than 2% (Fig 3 and S6 Fig). Next, we prepared M3-Oxa, G0-Oxa, G2-Oxa, and A2-Oxa as glycosyl donors of transglycosylation reactions, which have terminal mannose (Man), *N*-acetyl-glucosamine (GlcNAc), galactose (Gal), and *N*-acetylneuraminic acid (NeuAc) residues, respectively, and performed transglycosylation of their oxazoline to GlcNAc-anti-Her2 mAb using endoS-D233Q, as described previously [22]. The glycosylated rate of anti-Her2 mAbs from transgenic cocoons is 88.5%, and glycoengineered mAbs after transglycosylation consist of fully glycosylated, hemi-glycosylated and aglycosylated mAbs. Here, we performed an analysis and isolation of fully glycosylated mAbs from hemi-glycosylated mAbs using cation exchange column chromatography and obtained fully glycosylated homogeneous mAb-M3, mAb-G0, mAb-G2, and mAb-A2 (Fig 4). The size of *N*-glycan is approximately 4 × 3 × 1 nm, and *N*-glycan of mAbs occupies the Fc-Fc domain, but the entire conformation of the full-length antibody (antibody dimensions: approximately 15 × 10 × 5 nm [67]) is not affected by the *N*-glycan structure. However, the conformational variability and flexibility, which represent the distance between closed and open Fc-Fc structures, depend on the *N*-glycan structure of the mAb [7, 8, 68], and mainly affect the affinity of FcγR that mediates differential activity. Thus, we measured the affinity towards FcγRIIIa-V158 to investigate the use of *N*-glycans in glycoengineered mAbs. FcγRIIIa (CD16a) is a transmembrane glycoprotein expressed by NK cells and macrophages and a receptor for IgG, and its variant, V158 (FcγRIIIa-V158), shows a higher affinity than that of the F158 variant. FcγRIIIa and FcγRIIIa-V158 have five *N*-glycosylation sites (Asn-38, 45, 74, 162, and 169), and *N*-glycan at Asn-162 is critical for high affinity binding to the Fc domain based on the carbohydrate-carbohydrate interaction; this allows for discrimination between fucosylated and afucosylated IgG glycoforms due to steric hindrance [7, 8, 9, 10, 56, 57, 58]. Thus, we used recombinant human FcγRIIIa-V158 produced in HEK293 cells for the FcγRIIIa binding assay. As a result of the FcγRIIIa-binding assay (Fig 5), we observed the affinity of FcγRIIIa for glycoengineered anti-Her2 mAbs (mAb-M3, mAb-G0, mAb-G2, and mAb-A2), and confirmed the increasing affinities by defucosylation, the decreasing affinities by sialylation (mAb-G2 > mAb-A2), and the increasing affinities by neutral sugar extension (mAb-M3 < mAb-G0 < mAb-G2). Finally, we performed an ADCC reporter gene assay to investigate the function of *N*-glycans on therapeutic monoclonal antibodies (Fig 6), and found that the prepared homogeneous glycoengineered anti-Her2 mAbs (mAb-M3, mAb-G0, mAb-G2, and mAb-A2) have higher ADCC activity than that of anti-Her2 mAbs produced by CHO cells (trastuzumab) with a core fucose (S22 Fig). Although anti-Her2 mAbs with a terminal mannose residue (mAb-M3 and mAb consisting of mainly M5) show high potential for ADCC activity, these mAbs are not suitable as therapeutic antibodies, since they are cleared from the serum at a faster rate than other mAbs due to the high metabolism in the liver and spleen via the mannose receptor [69]. Thus, the *N*-glycan structure on mAbs may be able to target ADCC activity, which can be selectively active for target cells. Recently, antibody-drug conjugates (ADCs) have been developed as a targeted therapy for the treatment of cancer [70, 71, 72]. An ADC molecule consists of a cytotoxic drug and a tumor-targeting mAb or antibody fragment (single-chain variable fragment; scFv) that specifically binds to a specific tumor marker. One possible mechanism of ADCs is that the cytotoxic drug is released from the ADC and induces tumor killing after the internalization of ADC, which binds to a targeting marker on the tumor and is then internalized. Although the action of ADC occurs in the absence of effector cells, receptor-mediated endocytosis, which transports extracellular molecules into a target cell, is an important process for the action of ADCs. The evaluation of *N*-glycan on mAbs using our approach may lead to the elucidation of *N*-glycan function involving receptor-mediated endocytosis and may be useful for the development of more effective ADCs.

## Conclusions

We were able to prepare glycoengineered anti-Her2 mAbs (mAb-M3, mAb-G0, mAb-G2, and mAb-A2) from anti-Her2 mAbs owing to the purification of fully glycosylated mAbs and the construction of homogeneous glycosylated mAbs using a chemoenzymatic approach. Then we found that the glycan structure of mAbs affects their affinity towards FcγRIIIa-V158 and ADCC activity. In addition, we determined that the anti-Her2 mAbs (mAb, mAb-M3, mAb-G0, mAb-G2, mAb-A2) without a core fucose have higher ADCC activity than anti-Her2 mAbs produced by CHO cells (trastuzumab) with a core fucose in both SKBR-3 and BT-474 cells. This homogeneous glycosylated therapeutic mAb will provide insights into the effector functions, conformational changes or other pharmacokinetics (drug absorption, distribution, metabolism, and excretion) of IgGs at the molecular level and assist with rational engineering of therapeutic mAbs.

## Supporting Information

S1 Fig Cation-exchange HPLC analysis of a) anti-Her2 mAbs from CHO cells and b) anti-Her2 mAbs from silkworm cocoon (% is calculated from each fraction area for total area). Fully glycosylated mAbs, hemi-glycosylated mAbs and aglycosylated mAbs were assigned according to a previous study [39].
(TIF)

S2 Fig Mono S column chromatogram of anti-Her2 mAbs from silkworm cocoon (A). Cation-exchange HPLC analyses of fraction 1 (fully glycosylated anti-Her2 mAb) (B) and fraction 2 (C) isolated from Mono S column chromatogram in S2A Fig.
(TIF)

S3 Fig ESI orbitrap mass spectra of anti-Her2 mAb from silkworm cocoon (A) and fraction 1 (fully glycosylated anti-Her2 mAb) from S2 Fig (B). Deconvoluted spectra of anti-Her2 mAb from silkworm cocoon (C) derived from spectrum A, and fraction 1 (fully glycosylated anti-Her2 mAb) from S2 Fig (D) derived from spectrum B. Calcd value (monoisotopic mass: 147,524.8) was calculated from two heavy chains (1–449; 49,133.5) and two light chains (1–214; 23,428.5) and 16 disulfide bonds and two M5 glycans (1,216.4).
(TIF)

S4 Fig Overlapping MALDI QIT-TOF MS spectra of mAb glycopeptides with or without the ENG’ase (endoS) reaction. (TIF)

S5 Fig MALDI QIT-TOF MS spectra of mAb glycopeptides with or without the ENG’ase reaction (a; endoLL, b; endoH, c; endoD, d; endoM, e; endoS, f; no enzyme). (TIF)

S6 Fig SDS-PAGE of anti-Her2 mAb with or without the ENG’ase reaction (intact; no enzyme, S; endoS, LL; endoLL, H; endoH, D; endoD, M; endoM, D+S; endoD and endoS, D+LL+S; endoD and endoLL and endoS). (TIF)

S7 Fig 600 MHz 1H-NMR (D_2_O) of G0-OH. (TIF)

S8 Fig 600 MHz 1H-NMR (D_2_O) of G2-OH. (TIF)

S9 Fig 600 MHz 1H-NMR (D_2_O) of A2-OH. (TIF)

S10 Fig 600 MHz 1H-NMR (D_2_O) of M3-OH. (TIF)

S11 Fig 600 MHz 1H-NMR (D_2_O) of M3-Oxa. (TIF)

S12 Fig 600 MHz 1H-NMR (D_2_O) of G0-Oxa. (TIF)

S13 Fig 600 MHz 1H-NMR (D_2_O) of G2-Oxa (TIF)

S14 Fig 600 MHz 1H-NMR (D_2_O) of A2-Oxa. (TIF)

S15 Fig Stability of A2-Oxa under the acidic and neutral conditions. A2-Oxa (final concentration, 2.5 mM) was dissolved in 100 mM sodium phosphate buffer (pH 6.2–8.0). The decomposed ratio (A2OH/A2OH and A2Oxa) was monitored by MALDI-TOF MS in negative mode using α-cyano-4-hydroxycinnamic acid diethylammonium salt as the matrix at 0, 15, 30, 60, 120, 180, 300, and 480 min. A2-OH and A2-OXa were observed as *m/z* 2040.4 and 2022.7 [M+Na-2H]^-^, respectively. The decomposed ratio was calculated based on ion intensities.
(TIF)

S16 Fig SDS-PAGE analysis of transglycosylation using G2-Oxa as a donor substrate and GlcNAc-anti-Her2 mAb as an acceptor with the endoS-D233Q mutant at 0, 0.5, 1.0, 3.5, and 6 h. Translycosylated heavy chains with G2 *N*-glycans are shown as 54.1 kDa proteins, and unreacted aglycosylated heavy chains are shown as 52.5 kDa proteins. Light chains are shown as 29.8 kDa proteins.
(TIF)

S17 Fig SDS-PAGE analysis of anti-Her2 mAb from silkworm cocoon (lane intact), GlcNAc-anti-Her2 mAb from silkworm cocoon (lane acceptor), the transglycosylated anti-Her2 mAb with M3-OXa (lane M3), G0-Oxa (lane G0), G2-Oxa (lane G2), and A2-OXa (lane A2). The transglycosylated anti-Her2 mAbs were a mixture of fully glycosylated mAb, which consisted of two reacted glycosylated heavy chains and two light chains, and hemi-glycosylated mAb, which consisted of a single reacted glycosylated heavy chain and a single unreacted aglycosylated heavy chain and two light chains. The transglycosylated anti-Her2 mAbs migrates two bands of reacted glycosylated and unreacted aglycosylated heavy chains.
(TIF)

S18 Fig Mono S column chromatogram of transglycosylated anti-Her2 mAbs with A2-OXa (A). Cation-exchange HPLC analyses of the transglycosylated anti-Her2 mAbs with A2-OXa (B) and fraction 1 isolated from Mono S column chromatograph in S18A Fig (C).
(TIF)

S19 Fig Mono S column chromatogram of transglycosylated anti-Her2 mAbs with G2-OXa (A). Cation-exchange HPLC analyses of the transglycosylated anti-Her2 mAbs with G2-OXa (B) and fraction 1 isolated from Mono S column chromatograph in S19A Fig (C).
(TIF)

S20 Fig Mono S column chromatogram of the transglycosylated anti-Her2 mAbs with G0-OXa (A). Cation-exchange HPLC analyses of the transglycosylated anti-Her2 mAbs with G0-OXa (B) and fraction 1 isolated from Mono S column chromatograph in S20A Fig (C).
(TIF)

S21 Fig Mono S column chromatogram of the transglycosylated anti-Her2 mAbs with M3-OXa (A). Cation-exchange HPLC analyses of the transglycosylated anti-Her2 mAbs with G0-OXa (B) and fraction 1 isolated from Mono S column chromatograph in S21A Fig(C).
(TIF)

S22 Fig MALDI QIT-TOF MS spectrum of glycopeptides from anti-Her2 mAbs produced in CHO cells (trastuzumab). (TIF)

S1 Text Whole amino acid sequences of endoS_wild, endoS-D233Q, endoD, endoH, endoLL, and endoM. (TXT)
