# Supplementary figures and images for: Glycoengineered Monoclonal Antibodies with Homogeneous Glycan (M3, G0, G2, and A2) Using a Chemoenzymatic Approach Have Different Affinities for FcγRIIIa and Variable Antibody-Dependent Cellular Cytotoxicity Activities
Source: PLoS One. 2015 Jul 22;10(7):e0132848. doi: 10.1371/journal.pone.0132848 (PMC4511734; doi:10.1371/journal.pone.0132848)

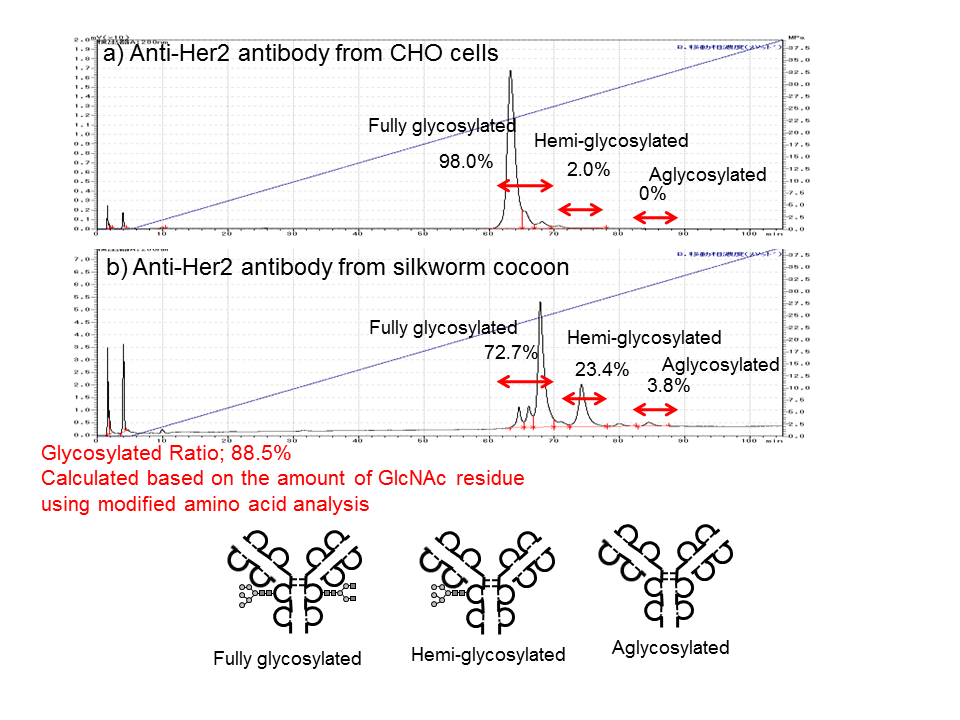

Supplement: S1 Fig — Fully glycosylated mAbs, hemi-glycosylated mAbs and aglycosylated mAbs were assigned according to a previous study [39]. (TIF) [file pone.0132848.s001.tif]

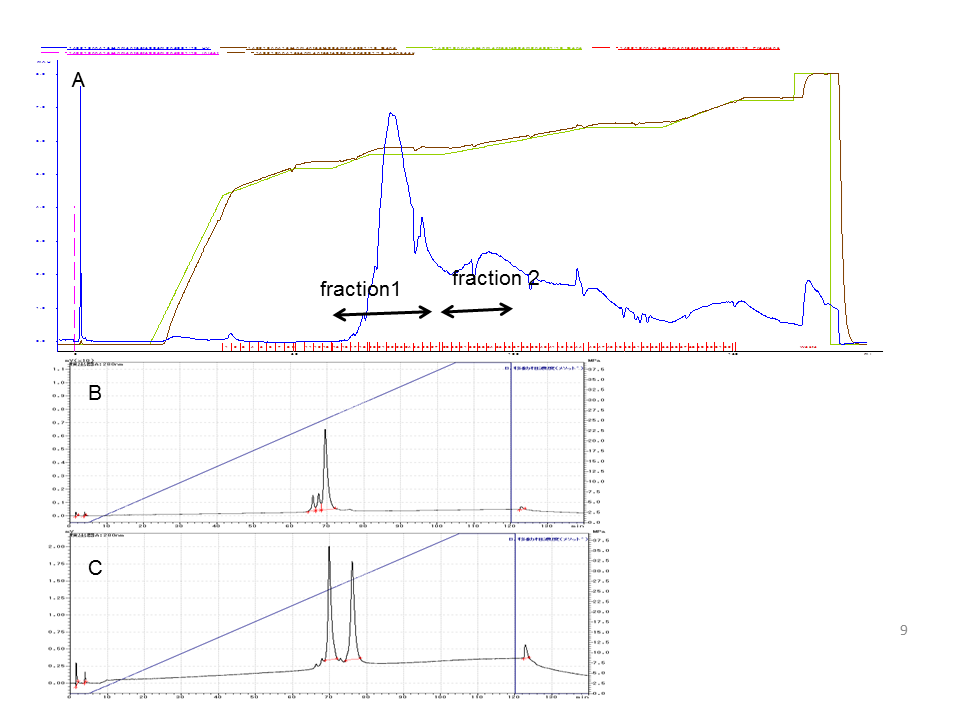

Supplement: S2 Fig — Mono S column chromatogram of anti-Her2 mAbs from silkworm cocoon (A). Cation-exchange HPLC analyses of fraction 1 (fully glycosylated anti-Her2 mAb) (B) and fraction 2 (C) isolated from Mono S column chromatogram in S2A Fig. (TIF) [file pone.0132848.s002.tif]

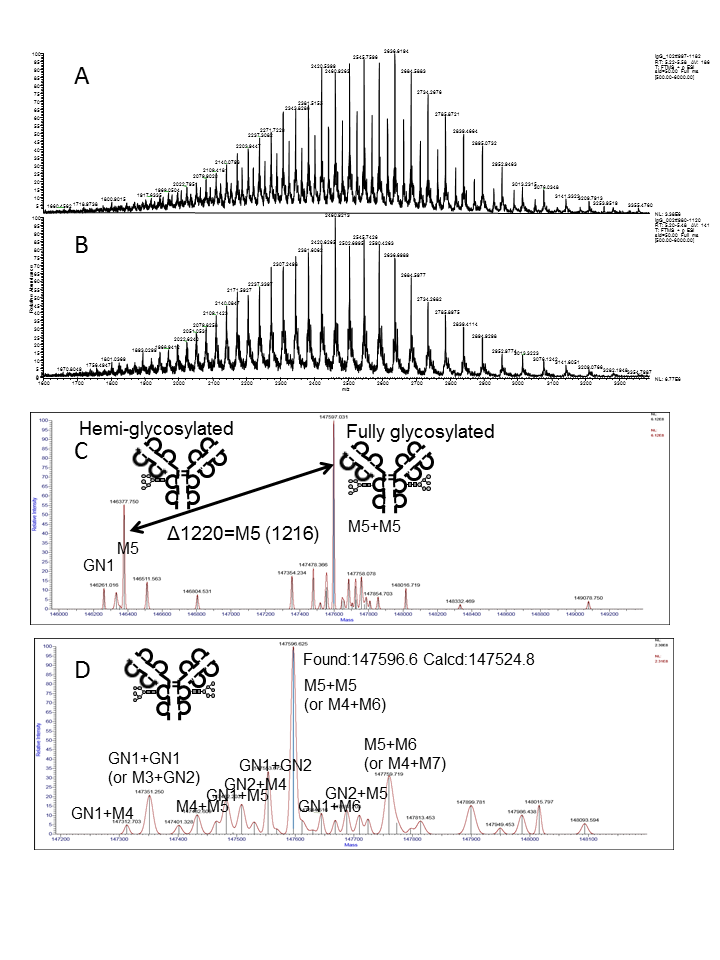

Supplement: S3 Fig — ESI orbitrap mass spectra of anti-Her2 mAb from silkworm cocoon (A) and fraction 1 (fully glycosylated anti-Her2 mAb) from S2 Fig (B). Deconvoluted spectra of anti-Her2 mAb from silkworm cocoon (C) derived from spectrum A, and fraction 1 (fully glycosylated anti-Her2 mAb) from S2 Fig (D) derived from spectrum B. Calcd value (monoisotopic mass: 147,524.8) was calculated from two heavy chains (1–449; 49,133.5) and two light chains (1–214; 23,428.5) and 16 disulfide bonds and two M5 glycans (1,216.4). (TIF) [file pone.0132848.s003.tif]

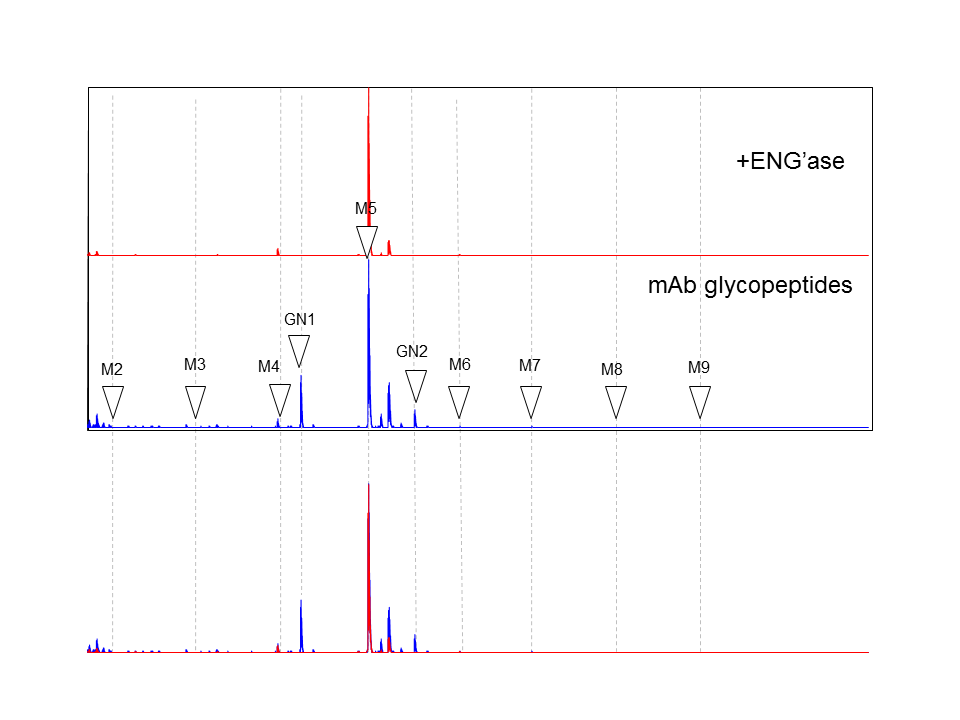

Supplement: S4 Fig — (TIF) [file pone.0132848.s004.tif]

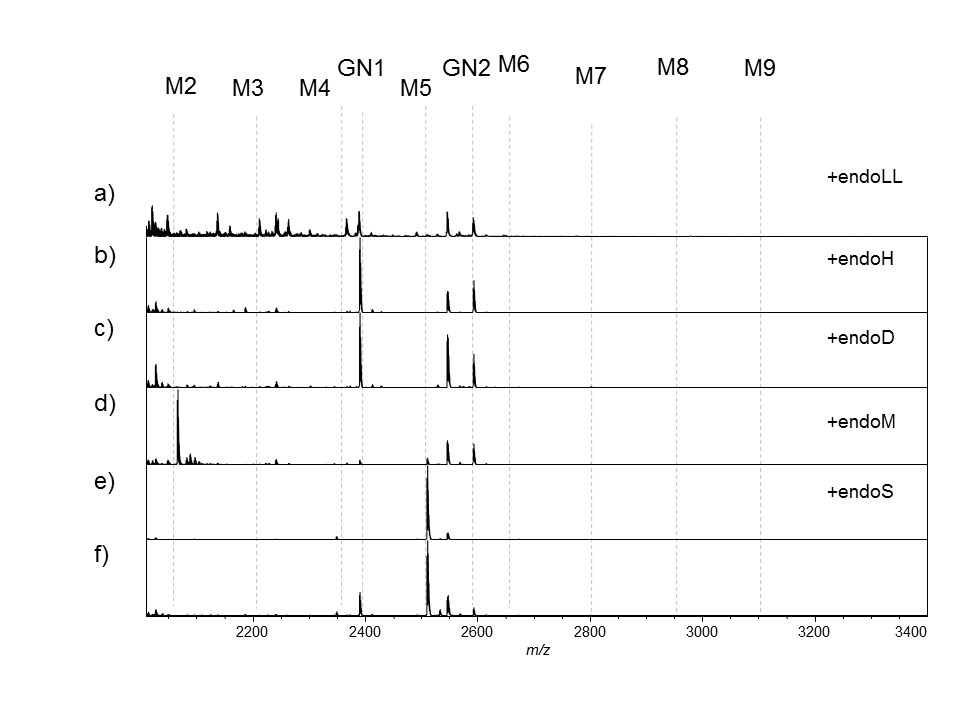

Supplement: S5 Fig — (TIF) [file pone.0132848.s005.tif]

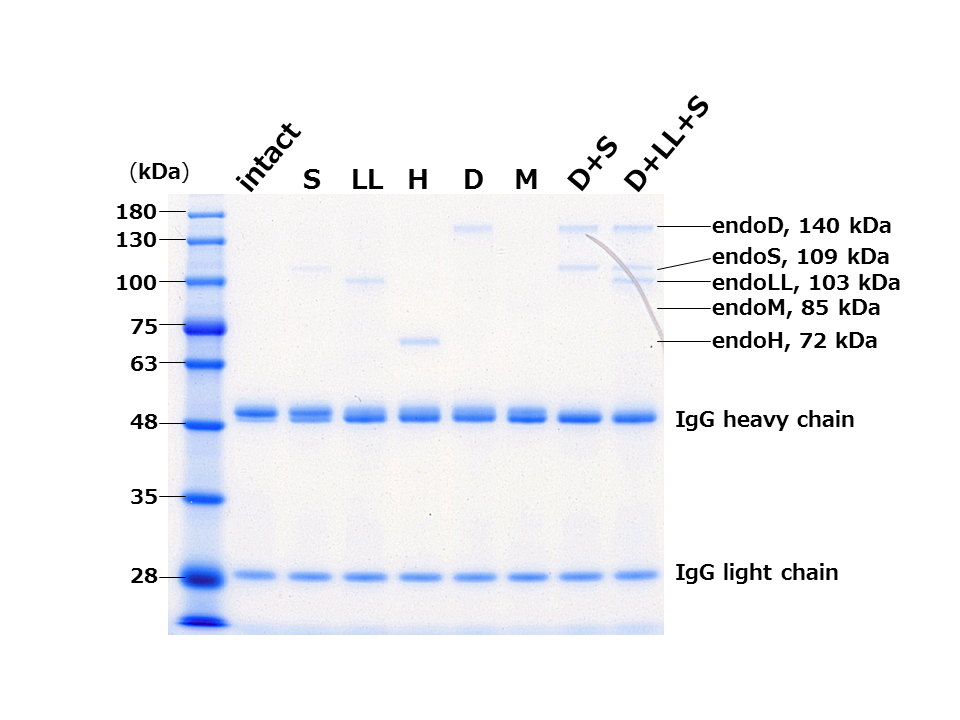

Supplement: S6 Fig — (TIF) [file pone.0132848.s006.tif]

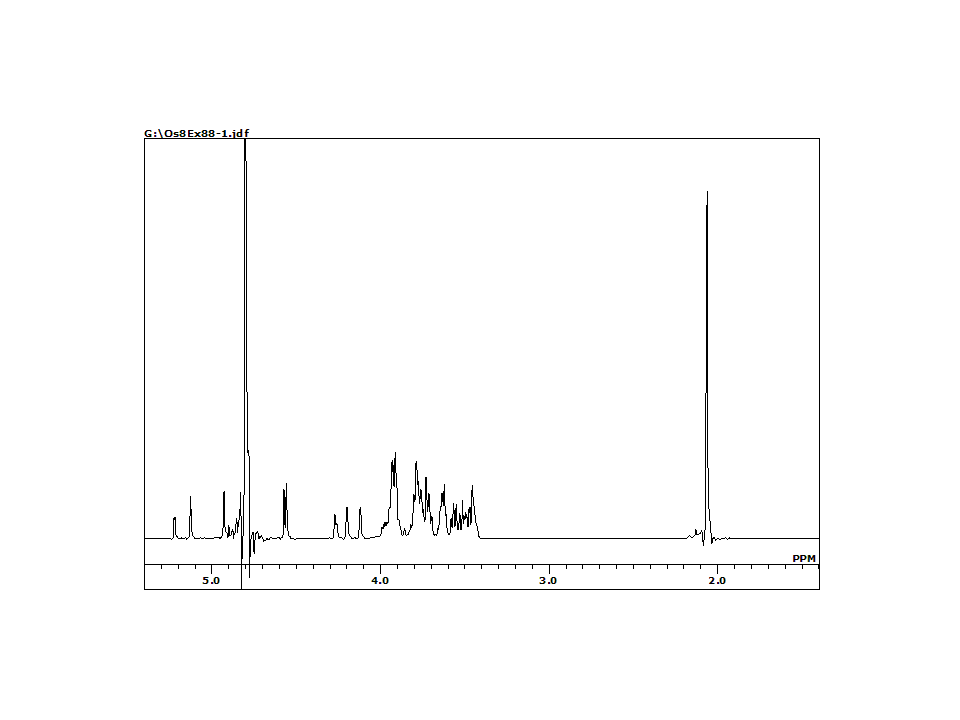

Supplement: S7 Fig — (TIF) [file pone.0132848.s007.tif]

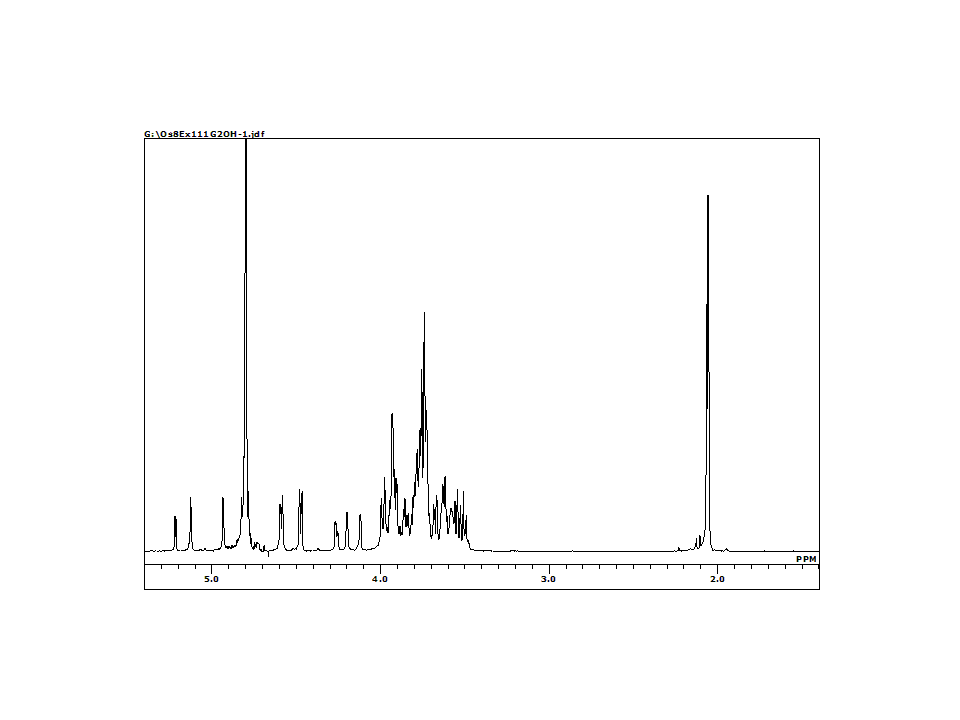

Supplement: S8 Fig — (TIF) [file pone.0132848.s008.tif]

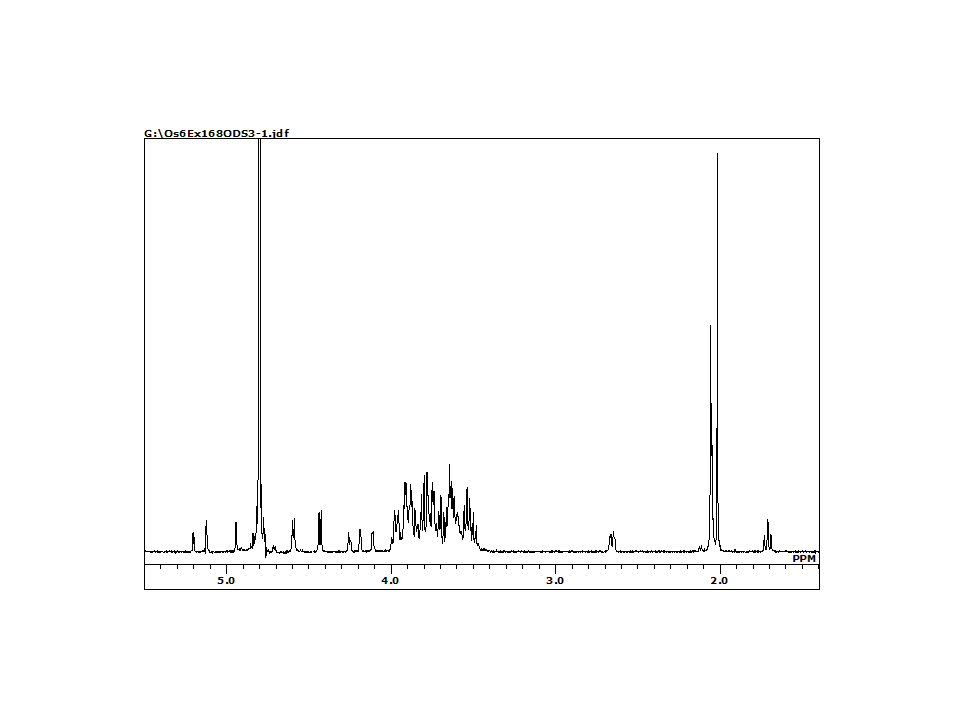

Supplement: S9 Fig — (TIF) [file pone.0132848.s009.tif]

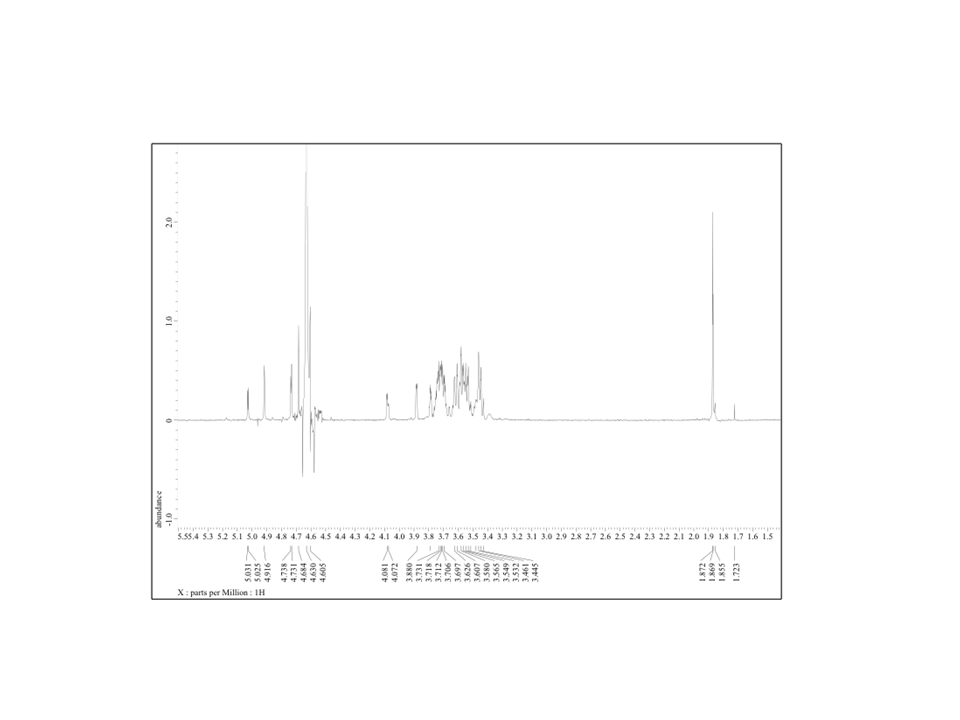

Supplement: S10 Fig — (TIF) [file pone.0132848.s010.tif]

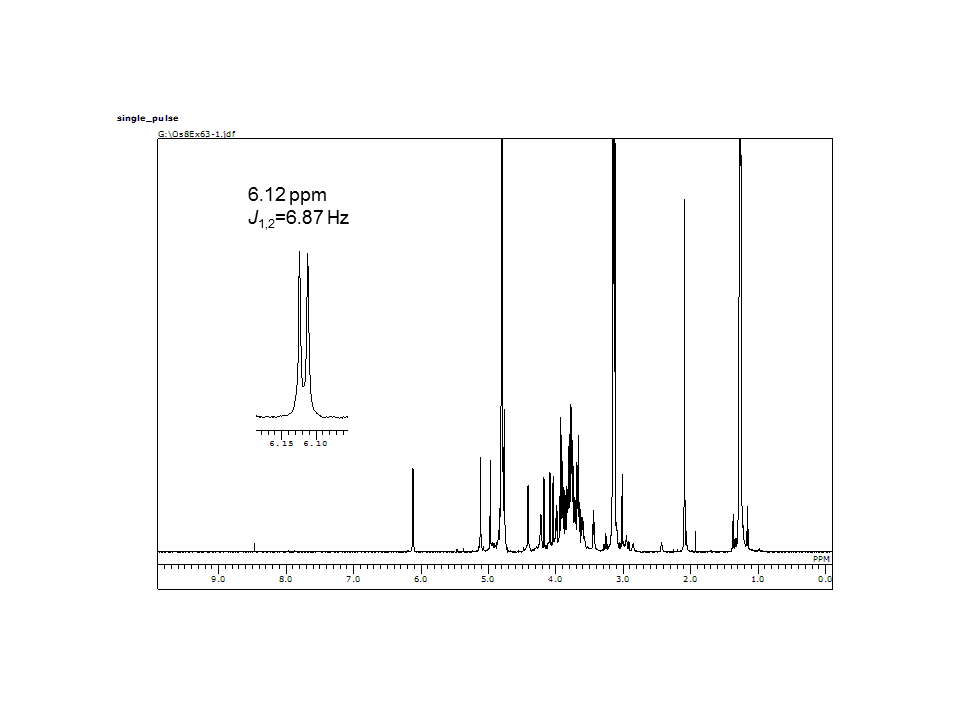

Supplement: S11 Fig — (TIF) [file pone.0132848.s011.tif]

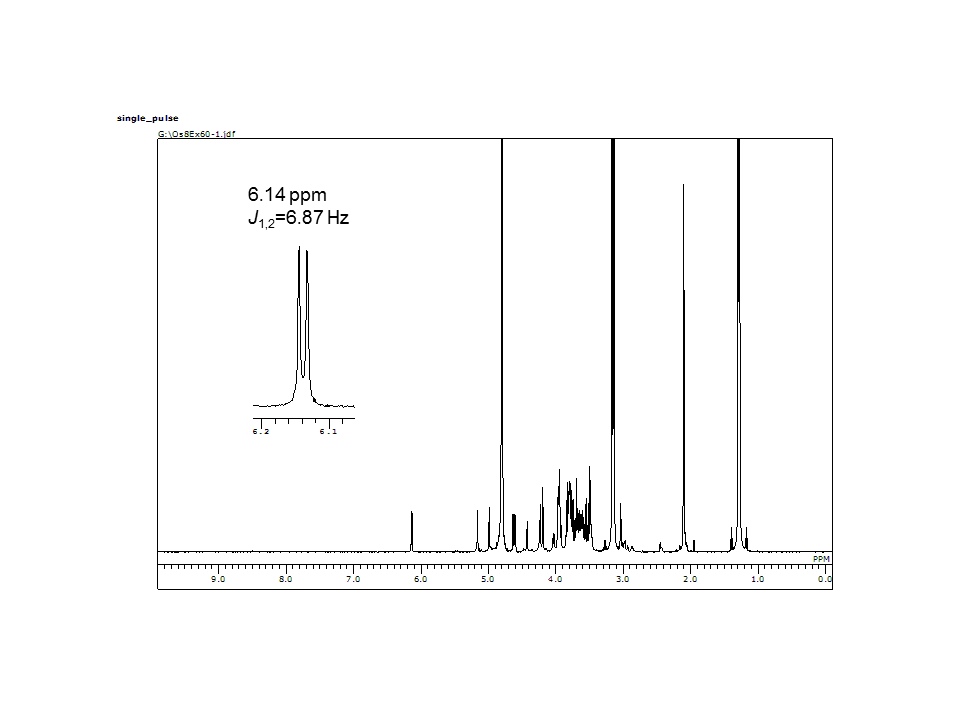

Supplement: S12 Fig — (TIF) [file pone.0132848.s012.tif]

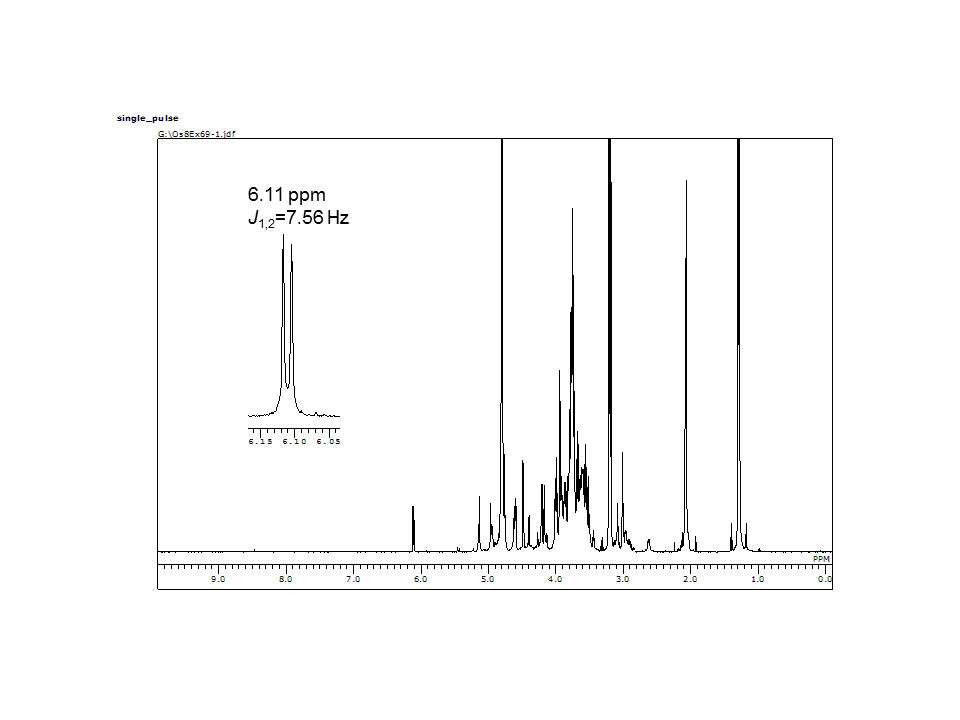

Supplement: S13 Fig — (TIF) [file pone.0132848.s013.tif]

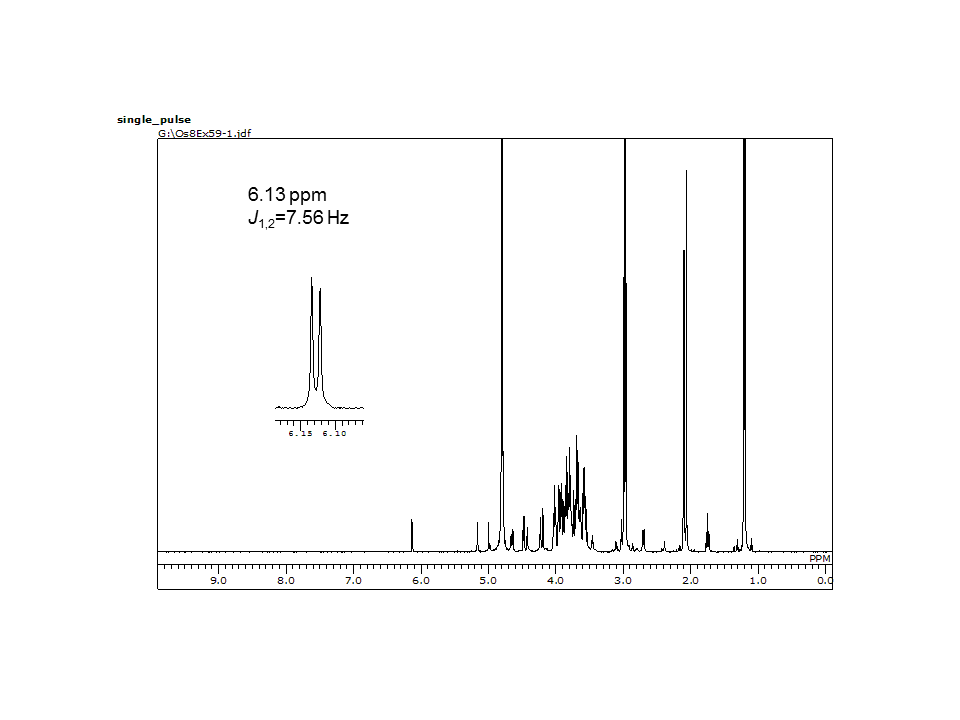

Supplement: S14 Fig — (TIF) [file pone.0132848.s014.tif]

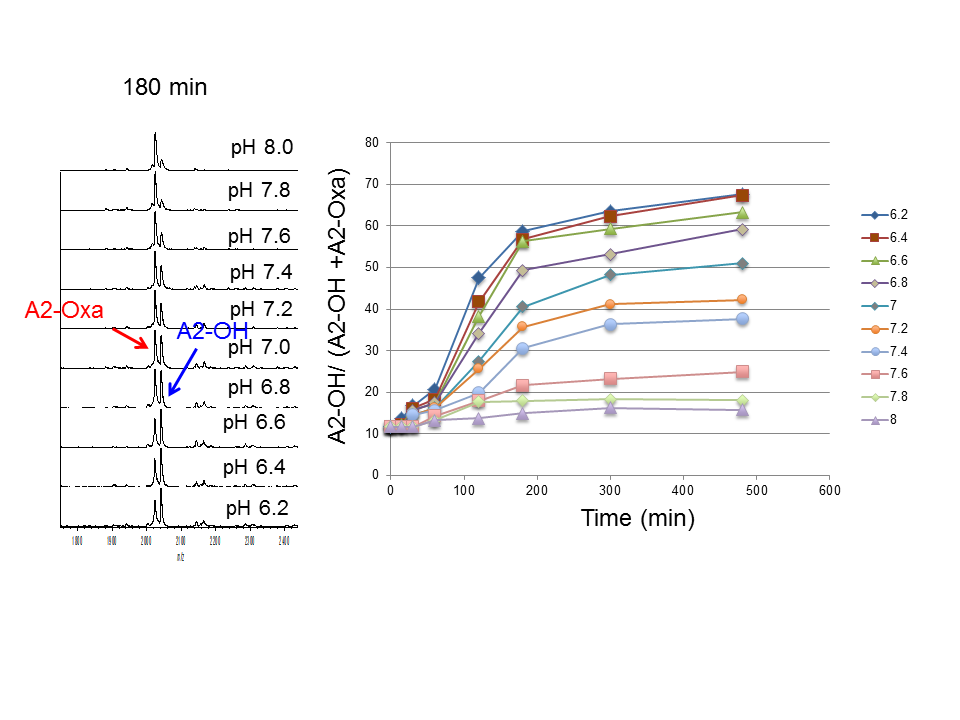

Supplement: S15 Fig — A2-Oxa (final concentration, 2.5 mM) was dissolved in 100 mM sodium phosphate buffer (pH 6.2–8.0). The decomposed ratio (A2OH/A2OH and A2Oxa) was monitored by MALDI-TOF MS in negative mode using α-cyano-4-hydroxycinnamic acid diethylammonium salt as the matrix at 0, 15, 30, 60, 120, 180, 300, and 480 min. A2-OH and A2-OXa were observed as m/z 2040.4 and 2022.7 [M+Na-2H]-, respectively. The decomposed ratio was calculated based on ion intensities. (TIF) [file pone.0132848.s015.tif]

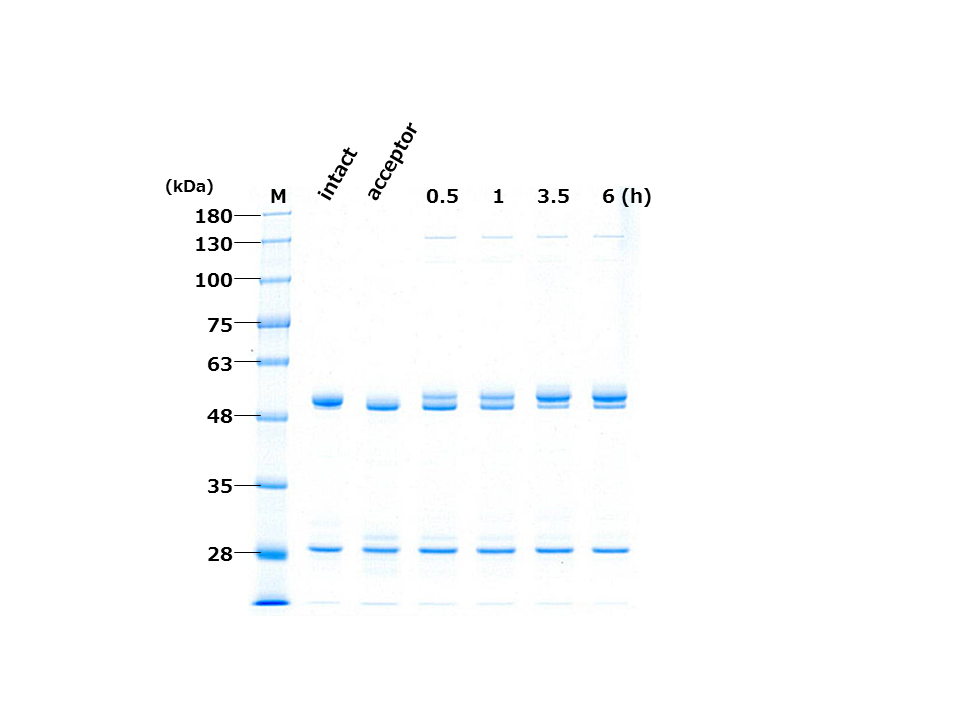

Supplement: S16 Fig — Translycosylated heavy chains with G2 N-glycans are shown as 54.1 kDa proteins, and unreacted aglycosylated heavy chains are shown as 52.5 kDa proteins. Light chains are shown as 29.8 kDa proteins. (TIF) [file pone.0132848.s016.tif]

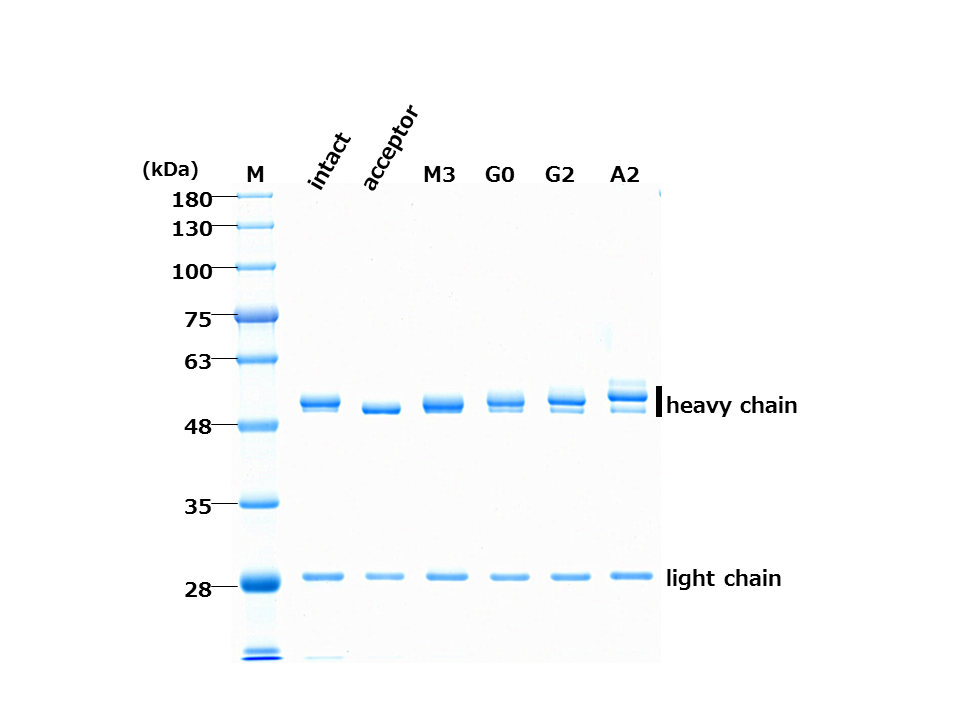

Supplement: S17 Fig — The transglycosylated anti-Her2 mAbs were a mixture of fully glycosylated mAb, which consisted of two reacted glycosylated heavy chains and two light chains, and hemi-glycosylated mAb, which consisted of a single reacted glycosylated heavy chain and a single unreacted aglycosylated heavy chain and two light chains. The transglycosylated anti-Her2 mAbs migrates two bands of reacted glycosylated and unreacted aglycosylated heavy chains. (TIF) [file pone.0132848.s017.tif]

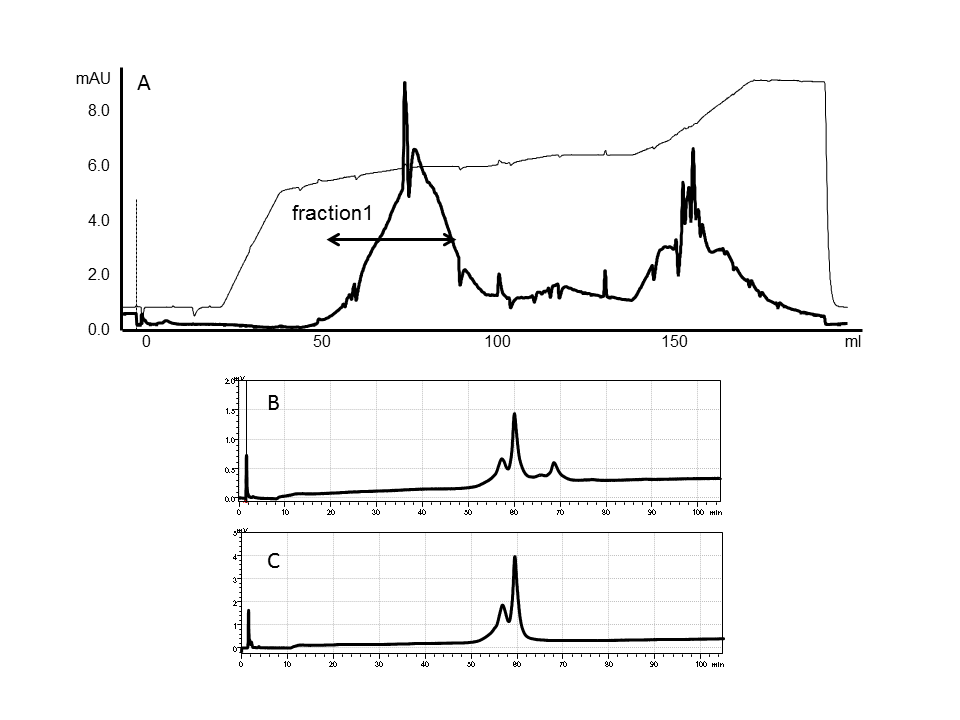

Supplement: S18 Fig — Mono S column chromatogram of transglycosylated anti-Her2 mAbs with A2-OXa (A). Cation-exchange HPLC analyses of the transglycosylated anti-Her2 mAbs with A2-OXa (B) and fraction 1 isolated from Mono S column chromatograph in S18A Fig (C). (TIF) [file pone.0132848.s018.tif]

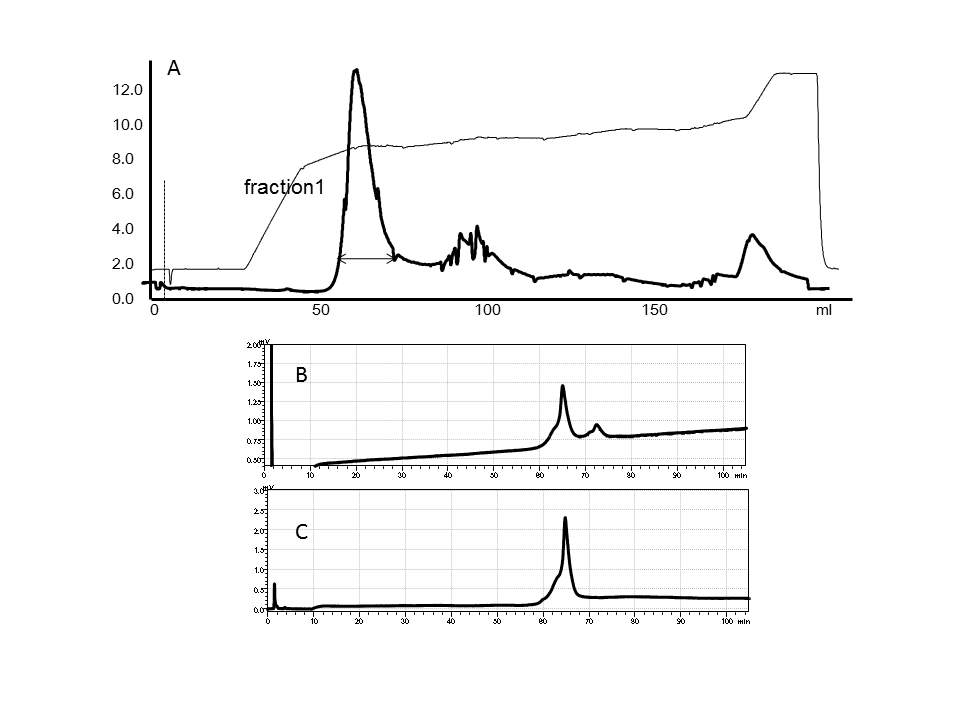

Supplement: S19 Fig — Mono S column chromatogram of transglycosylated anti-Her2 mAbs with G2-OXa (A). Cation-exchange HPLC analyses of the transglycosylated anti-Her2 mAbs with G2-OXa (B) and fraction 1 isolated from Mono S column chromatograph in S19A Fig (C). (TIF) [file pone.0132848.s019.tif]

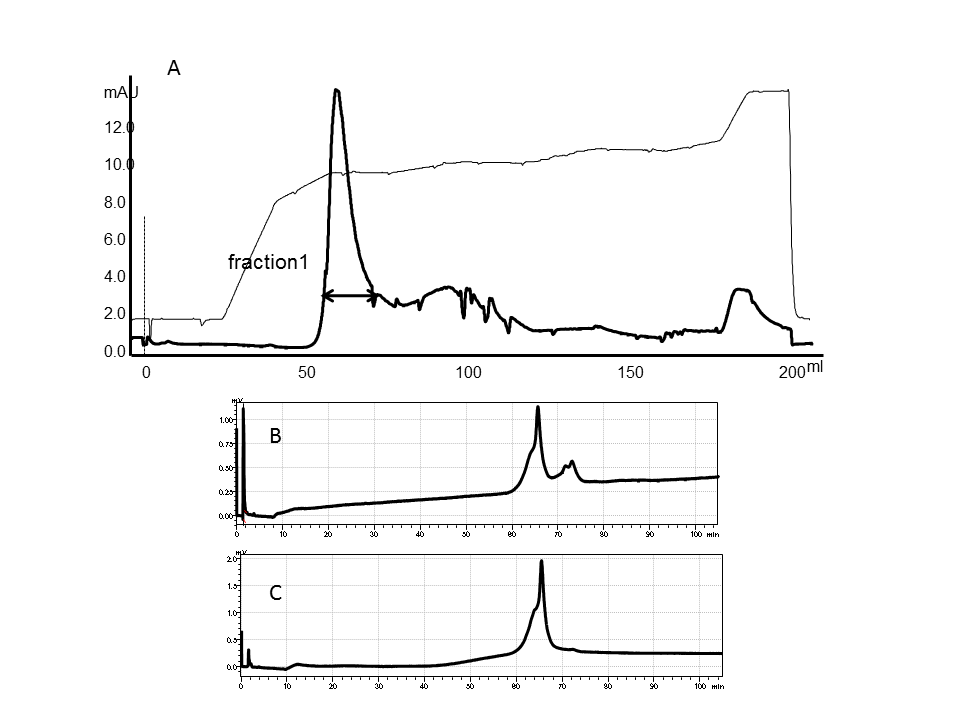

Supplement: S20 Fig — Mono S column chromatogram of the transglycosylated anti-Her2 mAbs with G0-OXa (A). Cation-exchange HPLC analyses of the transglycosylated anti-Her2 mAbs with G0-OXa (B) and fraction 1 isolated from Mono S column chromatograph in S20A Fig (C). (TIF) [file pone.0132848.s020.tif]

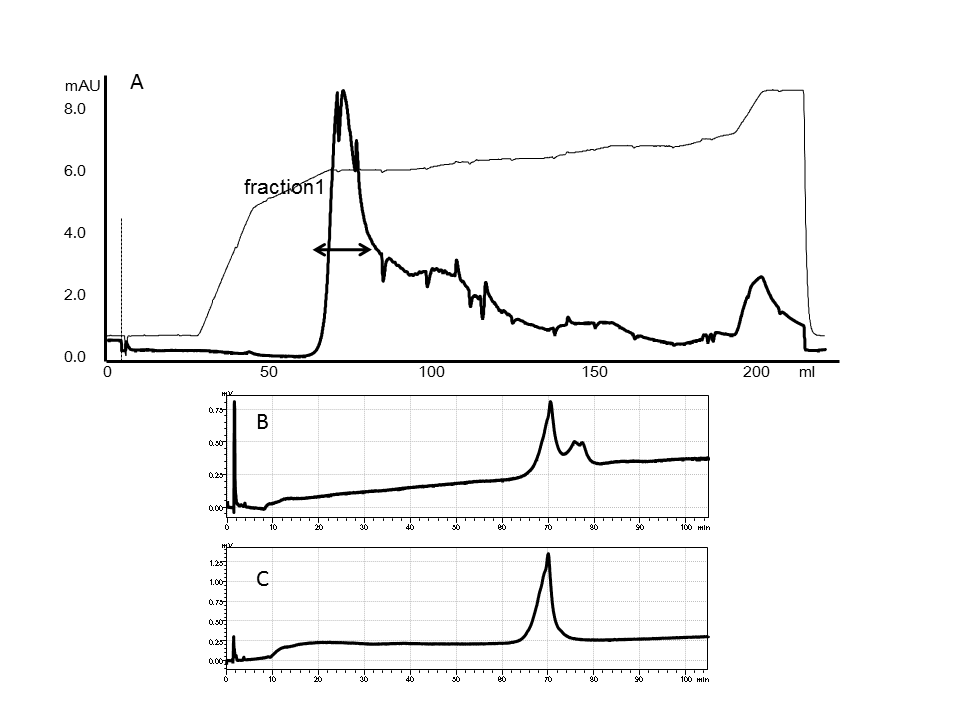

Supplement: S21 Fig — Mono S column chromatogram of the transglycosylated anti-Her2 mAbs with M3-OXa (A). Cation-exchange HPLC analyses of the transglycosylated anti-Her2 mAbs with G0-OXa (B) and fraction 1 isolated from Mono S column chromatograph in S21A Fig(C). (TIF) [file pone.0132848.s021.tif]

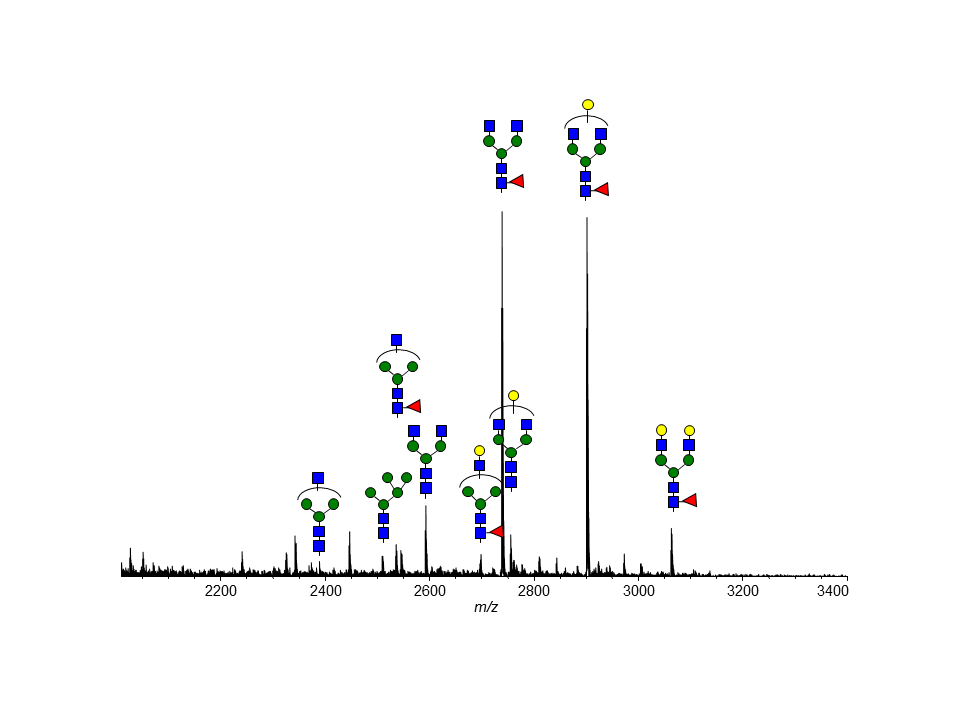

Supplement: S22 Fig — (TIF) [file pone.0132848.s022.tif]
